# New bioactive secondary metabolites from fungi: 2024

**DOI:** 10.1080/21501203.2025.2526772

**Published:** 2025-07-11

**Authors:** Meiyan Bao, Ying Shi, Xiaoyi Gong, Yutong Guo, Jing Wang, Xiaofei Chen, Ling Liu

**Affiliations:** a State Key Laboratory of Microbial Diversity and Innovative Utilization, Institute of Microbiology, Chinese Academy of Sciences, Beijing, China; b College of Life Sciences, University of Chinese Academy of Sciences, Beijing, China

**Keywords:** Fungal natural products, novel structures, chemical investigation, bioactivities, research strategies

## Abstract

Fungi have been recognised as a prolific source of structurally unique secondary metabolites with promising pharmacological properties. This review comprehensively summarises the chemical architectures, bioactivities, and research strategies for new fungal-derived natural products, focusing on representative studies published in 2024. A total of 907 novel fungal-derived compounds are systematically cataloged (isomers are also contained in the total count), including 284 polyketides, 362 terpenoids, 28 steroids, 108 alkaloids, 75 peptides, and 50 glycosides, while highlighting cutting-edge methodologies such as metabolomics-guided discovery platforms and biosynthetic pathway engineering. By establishing connections between chemical novelty and therapeutic value, this review not only provides strategic guidance for fungal chemical diversity exploration but also illuminates their transformative potential in overcoming drug discovery bottlenecks.

## GRAPHICAL ABSTRACT

**Figure:**
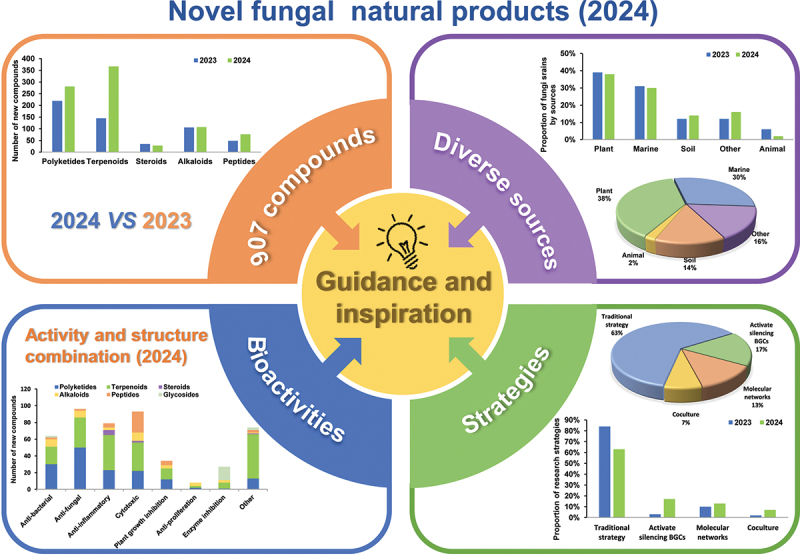

## 1. Introduction

This systematic review reveals a striking acceleration in the discovery of fungal-derived secondary metabolites, with 907 novel compounds (with 26 pairs of isomers counted in the total number) identified in 2024, with a 64.01% increase over the 553 compounds reported in 2023 (Shi et al. 2024c). This review does not provide a comprehensive listing of all compound structures, and only a selection of highlighted compounds (36 structures) is provided based on their chemical novelty, innovative characterisation methods, and potential biological activities. Consequently, these 907 compounds were classified into six structural categories, including terpenoids, polyketides, alkaloids, peptides, glycosides (a new class added compared to the previous year), and steroids. The numbering of compound isomers in this review follows the same scheme as the original article. Compound numbers marked with “*” denote unnamed structures (see Figure S1 for details), whereas those labelled with “#” indicate abbreviated labels for compounds with lengthy names (full names provided in Table S1).

**Figure 1.**
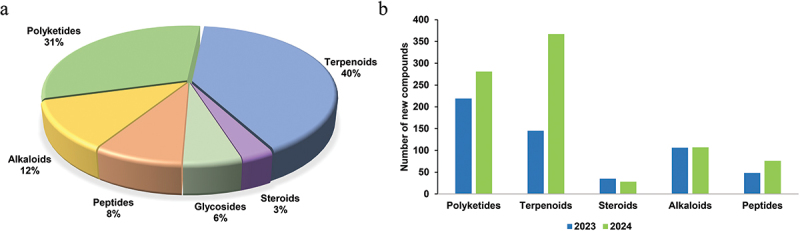
Number of novel fungal compounds in 2024. (a) Divided by structural types. (b) Comparison of structural types between 2023 and 2024.

**Figure 2.**
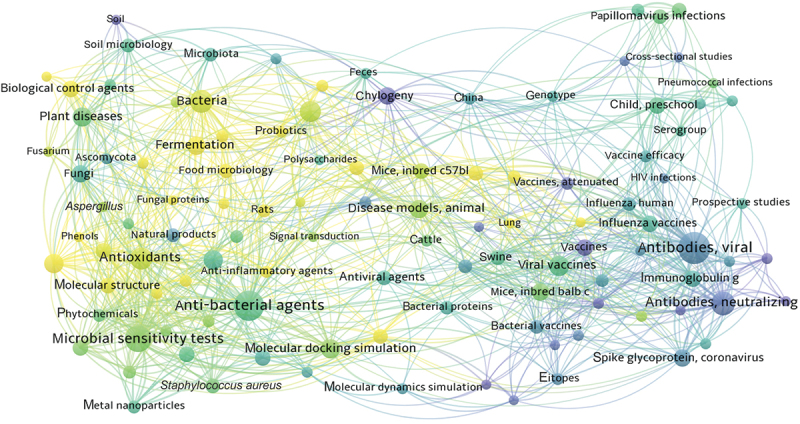
Pharmacology novel fungal-derived compounds (2024) by keyword co-occurrence network mapping analysis.

Structural categorisation showed that terpenoids were the most abundant class (40%, 362 compounds), followed by polyketides (31%, 284), alkaloids (12%, 108), peptides (8%, 75), glycosides (6%, 50), and steroids (3%, 28) (Figure 1a). The terpenoid group exhibited particularly explosive growth, with a 153.10% increase in discoveries compared to 2023, accounting for the majority of the overall expansion in fungal metabolite research (Figure 1b). As a continuation of the 2023 review (Shi et al. 2024c), this review maintains the narrative sequence of compound categories consistent with the prior publication. Additionally, using the core databases of Web of Science and PubMed as the sources, a keyword co-occurrence mapping was analysed by Visualization of Similarities (VOS) viewer (Figure 2). Analytical findings from the data reveal that the bioactivity studies of novel fungal-derived compounds in 2024 predominantly focus on antibacterial, antifungal, anti-inflammatory, and antioxidant activities. Investigations span both cellular and animal models, underscoring the robust potential of fungal natural products in drug lead compound discovery and the progressive refinement of pharmacological evaluations. Furthermore, molecular docking and dynamic simulation approaches are gaining broader application in mechanistic studies, while metagenomic technologies are undergoing significant methodological advancements.

In this review, we systematically summarise and analyse the structures, bioactivities, fungal strain sources, and research strategies of novel compounds, by conducting comparative analysis with 2023 data across multiple dimensions, which identifies evolving trends and highlights research hotspots, aiming to provide novel insights into the field of fungal natural products.

## 2. Chemistry and biological activities

### 2.1. Polyketides

Polyketides are synthesised through enzymatically catalysed Claisen condensation reactions. These reactions involve short-chain acyl initiators and elongation units such as acetyl-CoA, endowing polyketides with significant biochemical specificity and functional diversity. This section summarises 284 new polyketides isolated from fungi in 2024, including phenols, lactones, and aromas, etc.

**Figure 3.**
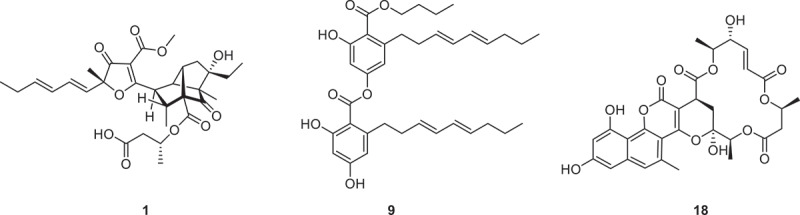
Structures of compounds **1**, **9**, and **18**.

Penidaleodiolides A and B (**1** and **2**), with “cage-like” structures, were isolated from a soil-derived fungus *Penicillium daleae* L3SO. Compound **1** features a novel tricyclo[4.3.0^4,9^]nonane moiety, a structural motif previously undocumented in polyketide-derived natural products (Figure 3). Electrophysiological analysis revealed that compound **1** dose-dependently inhibited hippocampal basket neuron action potentials and diminished sEPSC (spontaneous excitatory postsynaptic currents) frequency, while demonstrating no neurotoxicity (Wang et al. 2024d). The discovery of dicitrinols A–C (**3**–**5**) from *Penicillium citrinum* TW132–59 unveiled two unprecedented skeletons, including a 6/5/7/5 fused tricyclic framework and a topologically intricate cage-like decahydro-5,9,4-(epipropane[1,1,3]triyl)cycloocta[*b*]furan system (Wei et al. 2024). Genome mining techniques enabled the discovery of a previously unreported natural *ortho*-Quinone methides (*o*-QMs) precursor, designated trichophenol A (**6**), from the fungal strain *Trichoderma* sp. AT0167 (Wang et al. 2024j). Three phenolic compounds, carnemycin H (**7**), carnemycin I (**8**), and stromemycin B (**9**) (Figure 3), were found and identified from the marine-derived fungi *Aspergillus ustus*. Compound **8** demonstrated more potent antibacterial activity against *Ralstonia solanacearum* (MIC = 3 μg/mL, MBC = 30 μg/mL) than chloramphenicol (MIC = 10 μg/mL, MBC = 35 μg/mL) and rivalling streptomycin sulphate (MIC = 3 μg/mL, MBC = 30 μg/mL) in efficacy (Xue et al. 2024). From the marine-derived fungus *A. subversicolor* CYH-17, eight new metabolites compounds subversins A–E (**10**, **11a**/**11b**, **12**, **13**, **14a**/**14b**), subversic acid A (**15**), *epi*-wortmannine G (**16**), and 4-hydroxy-7-methoxyphthalide (**17**) were isolated (Che et al. 2024). Three novel natural products gymnoasins A−C (**18**−**20**) with unprecedented pentacyclic frameworks, were isolated from marine-derived fungus *Pseudogymnoascus* sp. HDN 17−895. These compounds represent the first natural products merging naphthopyrone and macrolide frameworks. The NLRP3 inflammasome is a potential therapeutic target for diseases such as gout, Alzheimer’s disease, and Parkinson’s disease. Biological assays have demonstrated that compound **18** (Figure 3) can significantly inhibit the activation of the NLRP3 inflammasome *in vitro* and effectively reduce the release of the pro-inflammatory cytokine interleukin-1*β* (IL-1*β*) *in* *vivo*, providing an important basis for the development of novel therapies that inhibit the NLRP3 inflammasome (Sun et al. 2024a).

Through regulation of bromine sources, 14 new brominated aromatic butenolides (compounds **21**^**#**^−**34**^**#**^) were isolated from the marine-derived endophytic fungus *A. terreus* EGF7-0-1. Compounds **21**^**#**^−**34**^**#**^ exhibited potent inhibitory activity against *Colletotrichum gloeosporioides* with EC_50_ values of 2.72−130.41 nmol/L, and were more effective than carbendazim (EC_50_ = 258.23 nmol/L) (Fan et al. 2024b). Heterologous expression of the *dpe* biosynthetic gene cluster from *Preussia isomera* XL1326 in *Saccharomyces cerevisiae* and *A. oryzae* chassis systems revealed a novel diphenyl ether derivative, compound **35**^**#**^ (Liu et al. 2024b). Researchers identified five new polyketides, including nigrosphaerone A–C (**36**–**38**), penivirone A (**39**), and penisulfoacid (**40**). Compound **36** was isolated from *Nigrospora lacticolonia* monoculture. Compounds **39** and **40** (with **40** being a novel sulphated derivative) originated from *Penicillium rubens* monoculture, while **37** and **38** were derived from *N. lacticolonia-P. rubens* coculture (Shi et al. 2024a).

**Figure 4.**
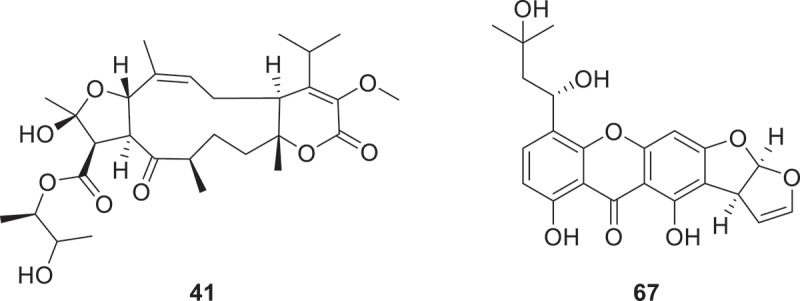
Structures of compounds **41** and **67**.

Seven new compounds stachybatranone A (**41a/41b**) and stachybatranones B−F (**42**−**46**) were isolated from *Sarcophyton subviride*. Compound **41** represented the first 5/11/6 fusion atranone compound containing a 2,3-butanediol group (Figure 4) (Lin et al. 2024). From phytopathogenic fungus *Stagonospora cirsii* Davis VIZR 1.41, a new 10-membered lactone (TML) named stagonolide L (**47**) was identified (Fedorov et al. 2024). From *Paraphaeosphaeria* sp. GU985234.1, researchers identified three new furan derivatives designated as paraketones A–C (**48**–**50**) along with an unprecedented polyketide named paraketone D (**51**). Simultaneously, investigations of *N. oryzae* KM513621.1 yielded two novel 10-membered macrolides (nigrosorlactones A and B, **52** and **53**), a linear polyketide variant designated as nigrosorlactone C (**54**), and the benzofuran-containing secondary metabolite cyclonigrotone A (**55**). Compounds **48**, **50**–**52**, and **54** demonstrated remarkable antiphytopathogenic efficacy against mycotoxigenic *Alternaria* sp. (MIC = 1 μg/mL), exhibiting potency equivalent to nystatin (MIC = 1 μg/mL) (Liao et al. 2024). Two previously unreported diastereomeric bicyclic lactones, brassictones A (**56**) and B (**57**), were successfully obtained through co-fermentation of the fungal strains *Alternaria brassicicola* and *Penicillium granulatum* (Tong et al. 2024). Ten novel derivatives designated as asperustins A–J (**58**–**67**) were obtained from *Aspergillus ustus* NRRL 5856 through bioactivity-directed isolation strategies. Remarkably, compounds **58**–**65** represent the inaugural members of the austocystin family characterised by an oxygen substitution at the C-4′ position, a structural novelty within this class of natural products (Chang et al. 2024a).

**Figure 5.**
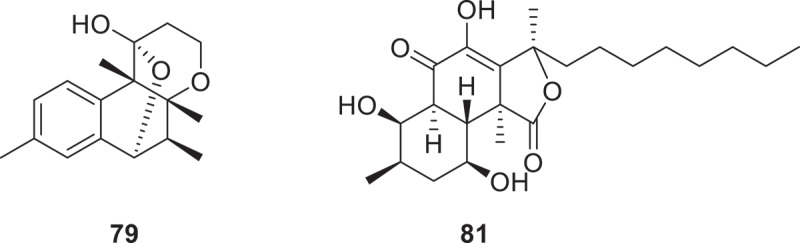
Structures of compounds **79** and **81**.

A novel secondary metabolite triorsellinaldehyde (**68**) was discovered in *A. nidulans* through targeted transcription factor engineering and overexpression. This approach successfully activated a previously silent biosynthetic gene cluster, leading to the production of the associated metabolic pathway (Rabot et al. 2024). Three aromatic derivatives tramevandins A−C (**69**−**71**) were obtained from the mixed fermentation of *Trametes versicolor* SY630 and *Vanderbylia robiniophila* SY341. Simultaneously, two additional metabolites 17-*ene*-1-deoxyPS (**72**) and 1-deoxyPS (**73**) were successfully isolated through the cocultivation. Structurally, **70** displays a novel carbon skeleton composed of a benzyl-substituted, highly modified *para*-terphenyl framework. Besides, compounds **72** and **73** exhibited potent antifungal activity against *Candida albicans* and *Cryptococcus neoformans* with MIC values of 3.13 μg/mL, demonstrating comparable efficacy to positive controls fluconazole, nystatin, and sphingosine (MICs = 6.25 μg/mL) (Ji et al. 2024). Seven novel compounds named aculeatiols A−G (**74**−**80**) were isolated from *A. aculeatus* BNCC 196238. Among them, compounds **74**–**76** were identified as stereoisomeric congeners, marking the inaugural identification of a *γ*-lactone moiety within the lovastatin-type chemical family. Furthermore, compound **79** emerges as the first characterised member of a previously uncharted 6/6/6/6-fused heterotetracyclic aromatic architecture (Figure 5) (Liu et al. 2024a). Fermentation of the plant endophytic fungus *Cladosporium cladosporioides* LD-8 led to the isolation of 14 previously unreported polyketides cladrioides A−S (**81**−**94**), with compounds **81** (Figure 5) and **82** uniquely incorporating a 6/6/5-fused tricyclic carbon skeleton. Significantly, compounds **87** and **90** exhibited superior antifungal activity against *Alternaria brassicicola* (IC_50_ = 7.42−9.28 μg/mL) and *Alternaria alternata* (8.96−9.21 μg/mL) to hymexazolcarbendazim (11.78 μg/mL and 28.2 μg/mL, respectively) (Han et al. 2024b).

Five novel polyketide compounds, including mycosporulonol (**95**), parasporulenones A−C (**96**−**98**) along with 10-norsporulenone (**99**) were isolated from the marine-derived fungus *Paraconiothyrium sporulosum* SFC20160907-M11. Compound **95** demonstrated stronger inhibition against *Phytophthora infestans* (MIC = 8 μg/mL) than the positive control (blasticidin S, MIC = 31 μg/mL) (Ngo et al. 2024). Integrated genomic-transcriptomic modulation was implemented to induce activation of the *A5cla* biosynthetic gene cluster in *Penicillium* sp. MYA5, leading to the structural characterisation of a novel compound designated as penicophenone F (**100**) (Sun et al. 2024c). Structural elucidation of marine-derived fungus *Peroneutypa* sp. M16 metabolites resulted in the identification of perochalasin A (**101**), perochalasin B (**102a**/**102b**), and perochalasin C (**103a**/**103b**) (de Amorim et al. 2024). Three novel depsidones identified as norflavicansone (**104**), flavicansone (**105**), and isocaloploicin (**106**) were isolated from *Teloschistes flavicans* TF-01. These hybrid structures uniquely combine orsellinic acid and rare configuration of *β*-orcinol moieties (Ferron et al. 2024).

**Figure 6.**
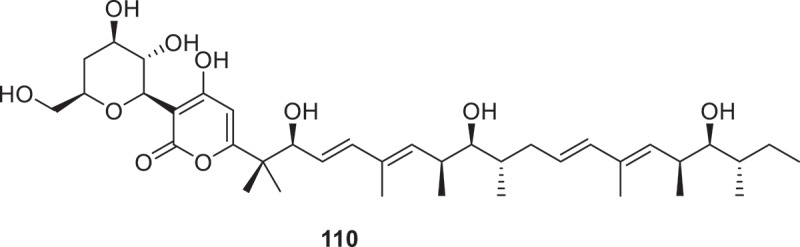
Structure of compound **110**.

Through genome mining, the investigators successfully isolated a pair of new enantiomers (±) -peniprenydiol A (**107a/107b**) from *Penicillium* sp. W21C371 (Wang et al. 2024b). Two novel benzoic acid derivatives were isolated from the endophytic fungus *Dentipellis fragilis* DSM 105465, named dentifragilone A (**108**) and dentifragilone B (**109**) (Sum et al. 2024). Three novel polyketide compounds identified as dactylfungin C (**110**), laburnicolin (**111**), and laburnicolenone (**112**) were isolated from *Laburnicola nematophila*. Compound **110** (Figure 6) exhibited superior antifungal efficacy against *Aspergillus fumigatus* ATCC 204305 (MIC = 0.26 μg/mL), with amphotericin B as the positive control (MIC = 0.31 μg/mL) (Wennrich et al. 2024b). Genome mining revealed an azaphilone biosynthetic gene cluster (*lut*) in the endophyte *Talaromyces* sp. XSJC-F4. Overexpression of the pathway-specific transcription factor *lutB* activated its silent pathway, therefore obtained seven novel sclerotiorin azaphilones (113^*^, 114a/b^*^, 115^*^, 116^*^, and 117a/b^*^) (Huang et al. 2024c). Co-cultivation of *Herpotrichia* sp. SF09 and *Trametes versicolor* SF09A induced metabolic interactions, leading to the isolation of two new compounds, hertramycines H (**118**) and I (**119**) (Wang et al. 2024o). Five new oxygenated *iso*-coumarins named setosphamarins A–E (**120**–**124**) were successfully isolated from the plant-derived fungal species *Setosphaeria rostrata* (Koopklang et al. 2024). Chemical investigation of *Rosellinia* sp. Glinf021 revealed eight previously unreported cytochalasans, rosellichalasins A–H (**125**–**132**) and two new shunt metabolites, rosellinins A and B (**133** and **134**) (Gu et al. 2024a).

**Figure 7.**
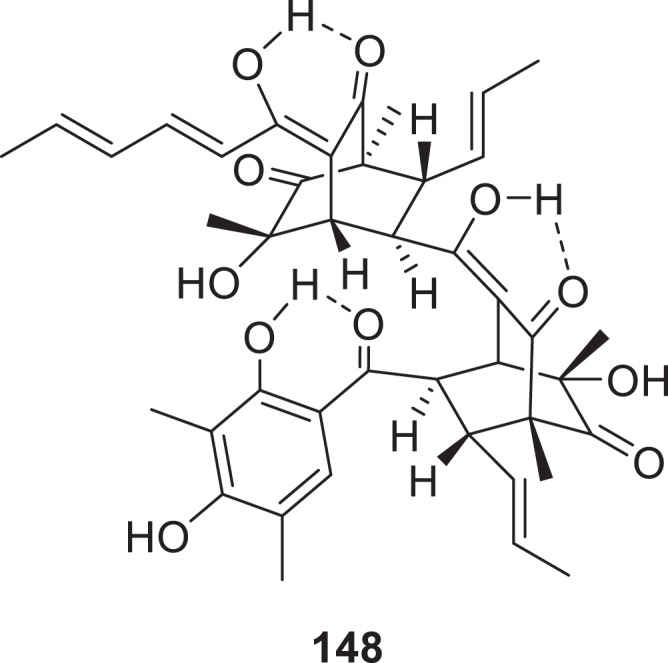
Structure of compound **148**.

Overexpression of the transcription factor gene 2642 activated a silent azaphilone biosynthetic gene cluster in the marine-derived fungus *Aspergillus terreus* RA2905. Metabolic profiling identified 11 novel compounds, including azasperones C–J (**135**–**142**) and preazasperones A–C (**143**–**145**) (Zheng et al. 2024d). Two novel *α*-pyrone derivatives, alternapyrones G and H (**146** and **147**), were isolated from the marine-derived fungus *Arthrinium arundinis* ZSDS-F3 (Hu et al. 2024). Three novel sorbicillinoid polyketides, designated citrinsorbicillins A–C (**148**–**150**), were identified from the endophytic fungus *Trichoderma citrinoviride* HT-9. Particularly, compound **148** represents the first trimeric sorbicillinoid reported from terrestrial fungi, featuring an unprecedented carbon skeleton formed through [4 + 2] (Diels-Alder-type) cycloaddition. This structural framework uniquely integrates two bicyclo[2.2.2]octanedione moieties bridged by an enolised methylene carbon (Figure 7) (Yin et al. 2024). A co-culture strategy employed between the endophytic fungi *Phomopsis asparagi* DHS-48 and *Phomopsis* sp. DHS-11 led to the isolation of a novel polyketide phomopyrone E (**151**) (Wu et al. 2024b).

Bioinformatic analysis of *D*-amino acid-activating adenylation domains led to the discovery of cordysetin A–C (**152**–**154**) from *Cordyceps militaris* ATCC 34164. Compound **152**, a novel tetramic acid antibiotic featuring a *trans*-decalin moiety, showed potent activity against *Mycobacterium tuberculosis* H37Rv (MIC = 0.016 µg/mL), surpassing rifampin (MIC = 0.25 µg/mL) (Gao et al. 2024). Two novel quinoline-orangeanin hybrids, penicilloneines A and B (**155** and **156**), were isolated from the marine-derived fungus *Penicillium* sp. GGF16-1–2, representing the first reported members of this hybrid class. Antifungal evaluation against *Colletotrichum gloeosporioides* via hyphal growth inhibition assays demonstrated potent activity for both compounds, with LD₅₀ values of 0.02 µg/mL (**155**) and 1.51 µg/mL (**156**), significantly surpassing carbendazim (49.58 µg/mL) and mancozeb (13.68 µg/mL) (Fan et al. 2024c). Four novel compounds *N*-isoamylsclerotiorinamine (**157**) and 7-methoxyl-*N*-isoamylsclerotiorinamine (**158**), along with two chromone derivatives, penithochromones X (**159**) and Y (**160**), were isolated from the endophytic fungus *Penicillium* sp. GDGJ-N37 (Huang et al. 2024a). Two novel polyketide compounds, named metguignardic acid (**161**) and (−)-*epi*-guignardone I (**162**), were isolated from the laurel forest endophytic fungus *Phyllosticta* sp. YCC4 strain (Díaz et al. 2024).

**Figure 8.**
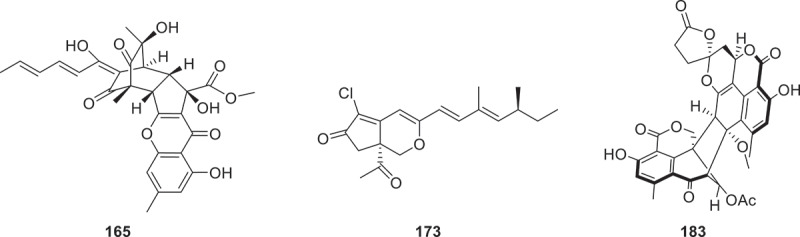
Structures of compounds **165**, **173**, and **183**.

Two new compounds were isolated and identified from Mariana Trench *Aspergillus* sp. SY2601, named 5-methoxy-8,9-dihydroxy-8,9-deoxyaspyrone (**163**), and 12*S*-aspertetranone D (**164**) (Sun et al. 2024b). Three unprecedented compounds named sorbremnoids A and B (**165** and **166**), sorbtalone (**167**) along with two additional novel compounds (*S*)-pyranaphthone and citrinoate (**168** and **169**), were isolated from the fungus *Penicillium citrinum* HDN11-186. Compounds **165** (Figure 8) and **166** are rare hybrid nanoparticles. They are generated by an MFS-like enzyme, which couples reactive intermediates from the *pcisor* and *pci*56 biosynthetic gene clusters (BGCs) (Zhang et al. 2024b). A new naphthalenoid pulvinic acid derivative (compound 170^*^) was isolated from *Pisolithus arhizus* (Parisi et al. 2024). Twelve new azaphilone analogs pensclazaphilones A−L (**171**−**182**) were isolated from the marine fungus *Penicillium sclerotium* UJNMF 0503. Compounds **173** (Figure 8) and **174** are the first azaphilones reported to feature a rearranged 5/6 bicyclic carbon framework coupled with a tetrahydropyran-modified side chain (Bao et al. 2024b). Taladuxins A−N (**183**−**196**) were isolated from the coral-derived fungus *Talaromyces* sp. TJ403-AL05. These compounds represent the first examples of duclauxin derivatives featuring a fused 1,6-dioxaspiro[4.5]decan-2-one moiety (**183**) (Figure 8), along with its biosynthetic transformation product (**184**), and 12 heptacyclic derivatives (**185**−**196**) containing a 6/6/6/5/6/6/6 ring system (Zeng et al. 2024a).

**Figure 9.**
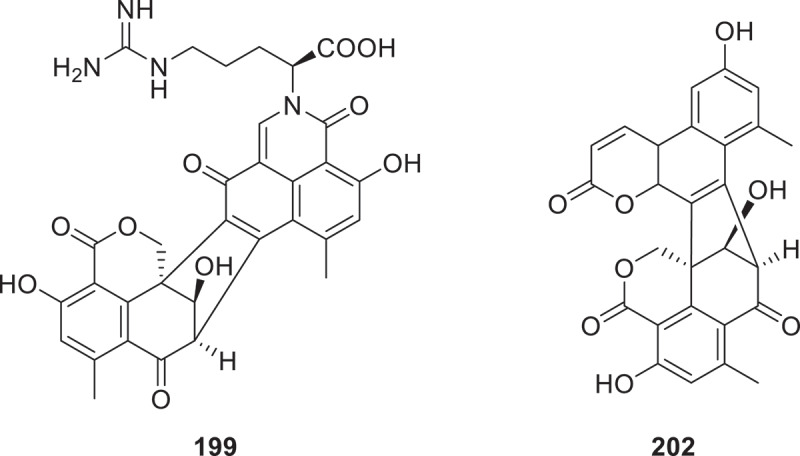
Structures of compounds **199** and **202**.

Genome mining and heterologous expression of the *sap* gene cluster in *Scedosporium apiospermum* F41-1 revealed two novel polyketide-amino acid conjugates, 2-*trans*-4-*trans*-2-methylsorbyl-*D*-leucine (**197**) and *N*-2-*trans*-4-*trans*-2-methylsorbyl leucenol (**198**). And compound **197** demonstrated potent inhibition of arabidopsis root growth (IC_50_ = 25 μmol/L), matching the efficacy of jasmonic acid methyl ester (25 μmol/L) (Chen et al. 2024b). Three secondary metabolites were isolated from the filamentous fungus *Talaromyces stipitatus*, which were identified as tealarohemiketal A (**199**), talaroazasone A (**200**), and talaromacrolactone A (**201**). Remarkably, compound **199** (Figure 9) features a unique oxybiotriphentenone dimer backbone, incorporating an unprecedented dioxytriphenphentenone hemiacetal moiety (Chaudhary et al. 2024). Nine new oligophenalenone dimers, named adpressins A−G (**202**−**210**), were isolated from the fungus *Talaromyces adpressus*. Adpressin A (**202**) represents the first duclauxin derivative with an unusual 6/6/6/5/6/6/6 ring system (Figure 9). Adpressins D (**205**) and E (**206**) feature a novel pyrrolidine ring (Zheng et al. 2024a).

Three new polyketides, paraphaeoketones A–C (**211**–**213**), were isolated from *Paraphaeosphaeria* sp. KT4192. Paraphaeoketone C (**213**) is a rare dimer of two paraphaeoketone B (**212**) units linked at C-10 (Oinuma et al. 2024). Seven novel *N*-containing oligophenolic dimers named talauromides A–G (**214**–**220**) were isolated from *Talaromyces stipitatus* BMC-16 (Zong et al. 2024). Employing a gene evolution-guided strategy, researchers uncovered a cryptic cytochalasin-like cluster (*sla*) in an Antarctic fungus *Simplicillium lamelliciola* HDN13430. Heterologous activation of this cluster resulted in the production of a novel compound named slamysin (**221**) (Wu et al. 2024c). Co-culturing *Alternaria brassicicola* with *Penicillium* sp. HUBU0120 facilitated the isolation of two pairs of enantiomers (±)-alterpyrones F (**222a**/**222b**) and G (**223a**/**223b**) along with two novel metabolites, granulahydeoate (**224**) and granulaone (**225**) (Kong et al. 2024). The marine-derived fungus *Aspergillus* sp. MCCC 3A00392 produced two monocyclic cyclopropane-containing metabolites, hamavellone C (**226**) and (+)-stagonospone A (**227**) (Yu et al. 2024a).

Six secondary metabolites, proliferic acids A−E (**228**−**232**) and proliferapyrone B (**233**), were identified from *Fusarium proliferatum* CGMCC 3.4710. Compound **228** demonstrated potent phytotoxicity through significant root growth inhibition in bioassays (Wang et al. 2024h). Lutelodiene A (**234**), a new metabolite, was characterised from *Aspergillus luteorubrus* MST-FP2246 (Arishi et al. 2024). Heterologous expression of the ascochin biosynthetic gene cluster (*asc* cluster) from *Paraphaeosphaeria* sp. SJC-F9 in the model fungus *A. nidulans* strain A1145 successfully generated metabolite 235^*^, an essential biosynthetic precursor for the natural product ascochin (Bai et al. 2024a). Heterologous expression of the *pyl* biosynthetic gene cluster from *Setosphaeria* sp. SCSIO 41009 in *A. nidulans* A1145 facilitated functional analysis, leading to the identification of key intermediates 236^*^ and 237^*^ essential for tetrahydropyrenophorol biosynthesis (Zhang et al. 2024c). Two new diepoxide polyketides chaetoketoics A (**238**) and B (**239**) were characterised from the culture extract of a deep sea-sediment derived fungus *Chaetomium globosum* SD-347 (Li et al. 2024f). Seven novel polyketides, including neofusarubins A–D (**240**–**243**) and fusofuranones A–C (**244**–**246**), were isolated from *Fusarium solani* via solid-state fermentation (Gao et al. 2024).

**Figure 10.**
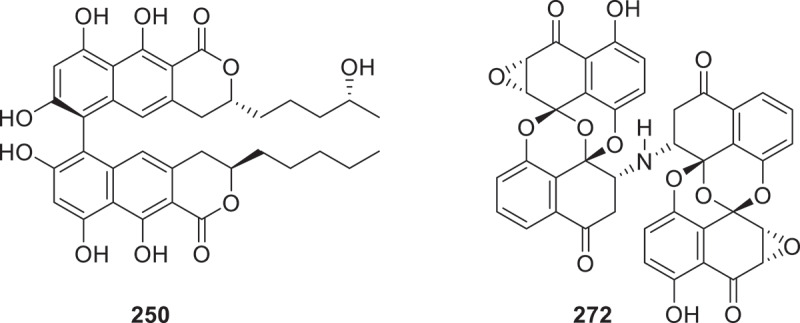
Structures of compounds **250** and **272**.

Coculture of *Monascus purpureus* and *A. oryzae* yielded two novel cyclohexyl-furans, monaspins A and B (**247** and **248**). Compound **248** showed potent anti-leukemic activity against HL-60 cells via apoptosis induction (IC_50_ = 160 nmol/L) (Meng et al. 2024b). Two novel secondary metabolites, ventiloquinone P (**249**) and talaroderxine D (**250**), were characterised from *Polyphilus sieberi*. Compound **250** (Figure 10) exhibited superior antimicrobial efficacy against *Bacillus subtilis* (MIC = 2.1 μg/mL), demonstrating a 7.9-fold enhancement over oxytetracycline (16.60 μg/mL) (Wennrich et al. 2024a). Three novel metabolites-two lactones, epiclactones A (**251**) and B (**252**), and a polyketide, epioxochromane (**253**)—were isolated from a co-culture of *A. oryzae* CGMCC 3.27588 and *Epicoccum dendrobii* YL001 (Shen et al. 2024a). Three novel dimeric xanthones, designated phomoxanthones L–N (**254**–**256**), were obtained through co-cultivation of *Phomopsis asparagi* DHS-48 with a congeneric fungal strain (DHS-11) (Wu et al. 2024b). A novel shikimate analog, methyl 5-*O*-acetyl-5-*epi*-shikimate (**257**) was isolated from the mangrove sediment-derived fungus *Roussoella* sp. SCSIO 41427 (Xiao et al. 2024). Four novel secondary metabolites, designated megastigmanones A–C (**258**–**260**) and prenylterphenyllin H (**261**), were obtained from the marine-derived fungus *Aspergillus* sp. ITBBc1 (Abulaizi et al. 2024). Peniapyrones A–E (**262**–**266**) with unprecedented 6,6,5-tricyclic scaffolds and peniapyrones F–I (**267**–**270**) containing novel 6,5,5-tricyclic *α*-pyrone frameworks were obtained from *Penicillium brefeldianum* F4a (Bai et al. 2024b). A previously uncharacterised tryptoquivaline analogue, 12*S*-deoxynortryptoquivaline (**271**), was isolated from the marine-derived fungus *Aspergillus clavatus* AS-107 (Doro-Goldsmith et al. 2024). Preussomerins N (**272**) (Figure 10) and O (**273**), characterised by intricate spirobisnaphthalene architectures, along with structurally simplified derivatives 2,3-*α*-epoxypalmarumycin CP18 (**274**) and 3*α*-hydroxy-CJ-12,372 (**275**), were isolated from the fungus *Roussoella* sp. KT4147. Compounds **272**–**275** exhibited potent growth inhibition against COLO-201 adenocarcinoma cells (IC_50_ = 5.4–9.8 μmol/L), significantly exceeding the activity of camptothecin (IC_50_ = 28.7 μmol/L) (Tokizaki et al. 2024).

### 2.2. Terpenoids

Terpenoids, structurally and biologically diverse isoprenoid derivatives, are classified according to their constituent isoprene units. This classification yields four principal categories: monoterpenes (10C, 2 units), sesquiterpenes (15C, 3 units), diterpenes (20C, 4 units), and triterpenes (30C, 6 units). This section compiles 362 distinct terpenoid structures spanning all major classes.

**Figure 11.**
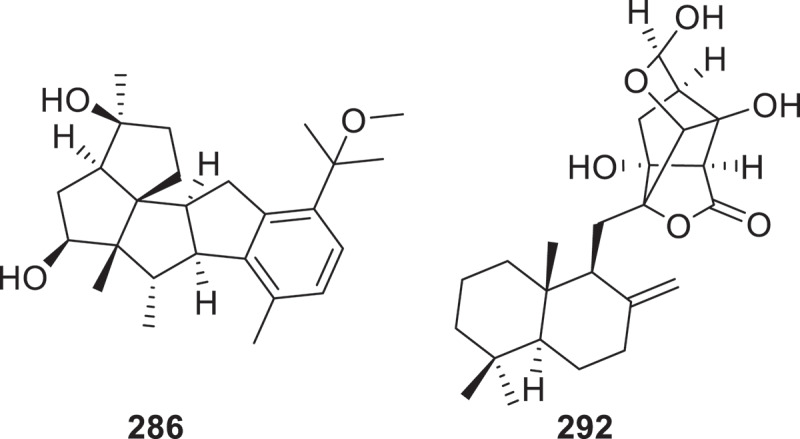
Structures of compounds **286** and **292**.

From the fungus *Helicoma septoconstrictum* TW03-2, four new drimane-type sesquiterpenoids, namely helicoitol A (**276**) and helicosides A–C (**277**–**279**), were identified (Zheng et al. 2024c). Six new meroterpenoids, penicianstinoids F−K (**280**–**285**), were isolated from the marine algicolous fungus *Penicillium* sp. RR-DL-1-7 (Miao et al. 2024). From the endophytic fungus *Aspergillus nidulans*, six novel sesterterpenoids named niduenes A−F (**286**−**291**) featuring an unprecedented 5/5/5/5/6 pentacyclic ring skeleton were isolated. Significantly, compounds **286** and **287** constitute the first reported aromatic pentacyclic sesterterpenoids (Fu et al. 2024a). The herqupenoid A (**292**) was a sesquiterpene-quinone hybrid with a rare 5/5/6/5-fused ring skeleton (Figure 11), and it was isolated from the soil-derived fungus *Penicillium herquei*. Compound **292** exhibited anti-inflammatory activity by inhibiting the NF-*κ*B-NLRP3 signalling pathway, with an IC_50_ value of 2.63 μmol/L, which was stronger than that of the positive control dexamethasone (IC_50_ = 6.00 μmol/L). Additionally, compound **292** showed no cytotoxicity at concentrations of 40 μmol/L (Jin et al. 2024).

The interaction of the host plant *Paris polyphylla* medium with microbial monocultures and cocultures led to the discovery of novel metabolites. These include eight sesquiterpenes, nigrophaphenoles A–G (**293**–**299**), featuring structural motifs distinct from canonical caryophyllene derivatives, and nigrophaphenone (**300**), a bicyclic compound with an unprecedented framework derived from *Nigrospora lacticolonia*. Additionally, five polyketide-terpenoid hybrids—penisulfoacid (**301**) and penisesketones A–D (**302**–**305**)—were identified from *Penicillium rubens* (Shi et al. 2024a). Two novel bipolyhydroindenol sesterterpenes, emerindanols A (**306**) and B (**307**), characterised by a 5/6–6/5 fused ring system, were isolated from the fungus *Emericella* sp. XL-029 (Liu et al. 2024c). Through genome mining, five novel meroterpenoids, farnesyltrypamides A–E (**308**–**312**), featuring a UbiA-type prenyltransferase (PTase)-mediated biosynthetic pathway, were identified from *Talaromyces variabilis* H1 (Yu et al. 2024b). Five new compounds identified as anthoteibinenes A–E (**313**–**317**) were obtained from the Irish deep-sea soft coral *Anthothela grandiflora*. These compounds are cadinene-like sesquiterpenes featuring an unusual dimethylamine substitution (Olsen et al. 2024). The fungus *Aspergillus xerophilus* produced a series of new compounds (**318**^#^–**324**^#^) which are classified as drimane-type sesquiterpenes (Salvatore et al. 2024). Three new eremophilane sesquiterpenoids paraphaterpene E–G (**325**–**327**) were isolated from *Paraphaeosphaeria* sp. Compound **327** (MIC = 1 μg/mL) showed antiphytopathogenic effects against mycotoxigenic *Alternaria* sp. comparable to the activity of nystatin (MIC = 1 μg/mL) (Liao et al. 2024).

**Figure 12.**
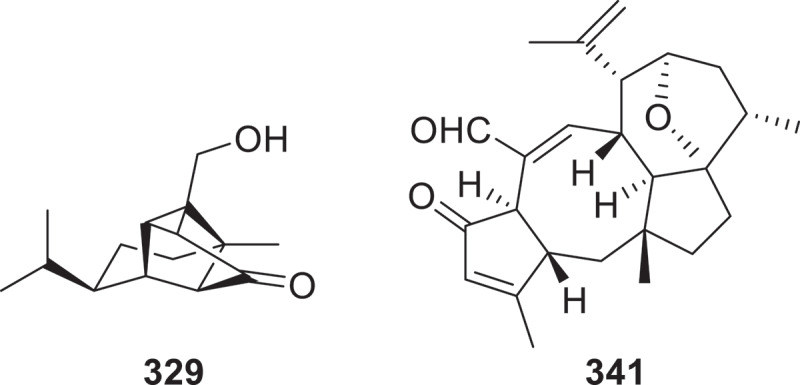
Structures of compounds **329** and **341**.

Four undescribed sativene-type sesquiterpenoids, namely bipolaric acid (**328**), bipolarone (**329**), bipolariol A (**330**), and bipolariol B (**331**), were isolated from the plant pathogenic fungus *Bipolaris sorokiniana* ACCC36805 by using a molecular networking strategy. Remarkably, compound **329** possesses a unique cage-like sesquiterpenoid skeleton (Figure 12), marking its first isolation from a fungal source, while compounds **330** and **331** feature a novel octahydro-1,4-ethanoisobenzofuran ring system (Wang et al. 2024n). Eight sesquiterpenes schizomycins A–H (**332**–**339**) and a meroterpenoid schizomycin I (**340**) were identified from *Schizophyllum commune* (Chen et al. 2024d). Five novel ophiobolin-derived sesterterpenoids, bipoladiens A−E (**341**–**345**), were isolated from the phytopathogenic fungus *Bipolaris maydis*. Compound **341** features a undescribed tetracyclic 5/8/5/7 fused carbon skeleton (Figure 12) (Shen et al. 2024b).

**Figure 13.**
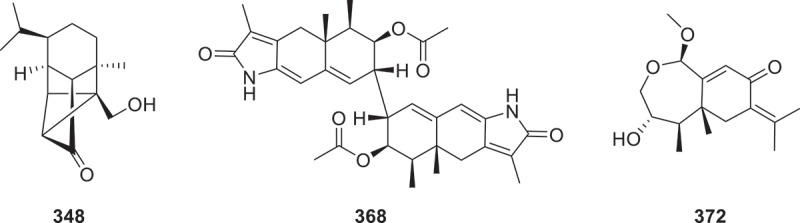
Structures of compounds **348**, **368**, and **372**.

From the marine fungus *Aspergillus terreus* SCSIO 41202, two compounds, asperporonins A (**346**) and B (**347**), have been isolated. These compounds are classified as caryophyllene-type sesquiterpenoids and possess a unique structural skeleton marked by the incorporation of two pyranoid rings. In antimicrobial assays against *Xanthomonas citri* subsp. *citri* (*Xcc*), both compounds showed equivalent antibacterial potency to copper sulphate, with identical MIC values of 0.3125 mg/mL (Zhang et al. 2024a). New sativene sesquiterpenoids bipotivene A (**348**) and bipotivene C−K (**349**−**360**), a new metabolite bipotivene B (**361**), were isolated from the endophytic fungus *Bipolaris victoriae* S27. Bipotivene A (**348**) was the first example of sativene sesquiterpenoids with a unique 6/5/3/5-caged tetracyclic ring system (Figure 13). Compound **349** significantly suppressed *Arabidopsis thaliana* growth, displaying a fresh weight inhibition rate of 7.96% ± 1.30%, while the commercial herbicide glyphosate showed a markedly higher inhibition rate (41.51% ± 1.41%) (Wang et al. 2024i). Bioguided isolation of the cultural broth of *T. laricinum* produced four new drimane sesquiterpenes, trichalarins A−D (**362**−**365**) (Huang et al. 2024b). Two new sesquiterpenoid derivatives, elgonenes M (**366**) and N (**367**) were isolated from the mangrove sediment-derived fungus *Roussoella* sp. SCSIO 41427 (Xiao et al. 2024). Molecular networking-guided isolation of the fungus *Penicillium roqueforti* led to the discovery of five previously undescribed eremophilane sesquiterpenoids, dipeniroqueforins A and B (**368** and **369**) and peniroqueforins B–D (**370**–**372**). Compounds **368** (Figure 13) and **369**, featuring an unprecedented lactam-type sesquiterpenoid scaffold with a homodimeric 5/6/6–6/6/5 hexacyclic system, constitute the first reported lactam-containing eremophilane derivatives. Compound **372** is identified as the first 1,2-*seco* eremophilane sesquiterpenoid bearing a novel 7/6-fused bicyclic framework (Figure 13) (Mo et al. 2024).

**Figure 14.**
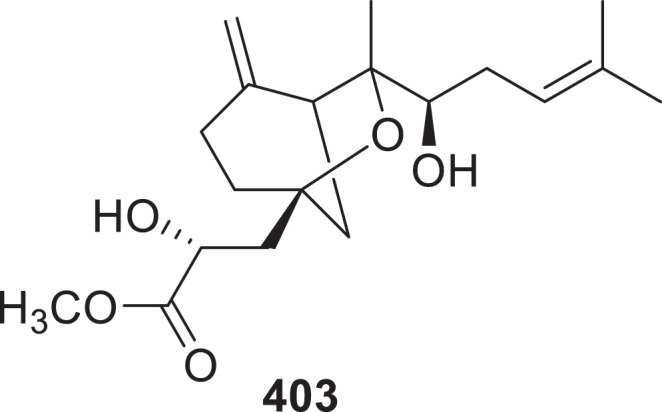
Structure of compound **403**.

Novel compounds breviane spiroditerpenoids (**373**^#^–**378**^#^) and carotane sesquiterpenoids (**379**^#^, and **380**^#^) were isolated from an endophytic fungus *Penicillium bialowiezense* ZBWPQ-27 (Chen et al. 2024c). Three nor-sesquiterpenes phellinharts A−C (**381**−**383**) were isolated from *Phellinus hartigii* and exhibited unprecedented protoilludane and cerapicane-type structures (Fu et al. 2024b). Acremonides A−G (**384**−**390**) were isolated from the cultures of a sponge-associated fungus, *Acremonium* sp. IMB18-086, which was cultivated with heat-killed *Pseudomonas aeruginosa* (Hao et al. 2024). The new compounds, medullins A−H (**391**−**398**), were isolated from the culture broth of *Perenniporia medulla-panis* (Ki et al. 2024). Co-cultivation of isopod-associated fungi *Herpo-trichia* sp. SF09 and *Trametes versicolor* SF09A led to the reciprocal induction of five new sesquiterpenes hertramycine C (**399a**/**399b**), D−F (**400**–**402)** (Wang et al. 2024o). Ten new sesquiterpenoids, pseuboyenes A–J (**403**–**412**), were isolated from the cold-seep-derived fungus *Pseudallescheria boydii* CS-793. Compound **403** represents the first *β*-bergamotene analogue incorporating a methyl lactate-conjugated 6-oxobicyclo[3.2.1]octane carbon skeleton (Figure 14). Compound **406** exhibited potent antifungal activity against *Coniothyrium cornigerum* (MIC = 4 μg/mL), *Fusarium oxysporum* (MIC = 2 μg/mL), and *F. proliferatum* (MIC = 0.5 μg/mL), surpassing the positive control amphotericin B (MIC = 8, 4, and 1 μg/mL, respectively) (Ying et al. 2024). Twelve novel ophiobolin-type sester-terpenoids, undobolins A−L (**413**−**424**), were isolated from *Aspergillus undulate* (Zheng et al. 2024d, 2024e).

**Figure 15.**
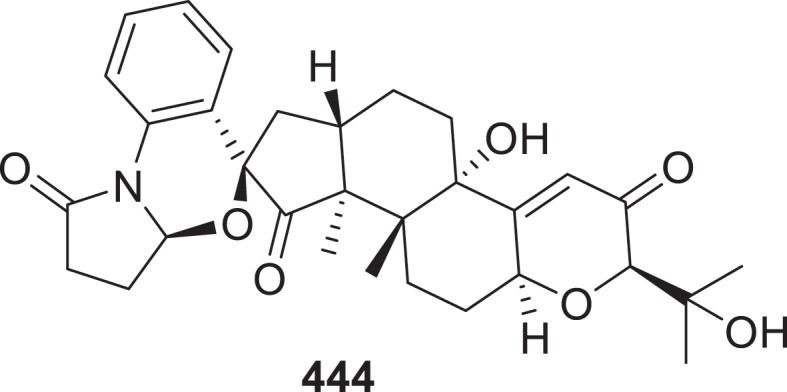
Structure of compound **444**.

The new compounds, oxybergamotenes A−H (**425**−**432**) were isolated from the marine-derived fungus *Trichoderma erinaceum* F1-1. The discovery was made through genome mining and heterologous expression in the host *A. nidulans* LO8030 (Yang et al. 2024b). Two 19-nor-diterpenoids, talascortenes H (**433**) and I (**434**), two diterpenoid acids, talascortenes J (**435**) and K (**436**), and one triterpenoid, talascortene L (**437**) were isolated from the sea-anemone-derived endophytic fungus *Talaromyces scorteus* AS-242 (Wang et al. 2024m). Three novel oxoindolo diterpenoids, emeniveols B–D (**438**–**440**), along with three bisabolane sesquiterpenoids—aspergol A (**441**), expansol H (**442**), and aspergol B (**443**)—were isolated and identified from the deep-sea-derived fungus *Aspergillus* sp. MCCC 3A00392 (Yu et al. 2024a). Seven new indole-diterpenoids penpaxilloids A–E (**444**–**448**), 7-methoxypaxilline-13-ene (**449**), and 10-hydroxy-paspa line (**450**) were isolated from the marine-derived fungus *Penicillium* sp. ZYX-Z-143. PTP1B represents a promising therapeutic target for type 2 diabetes mellitus. Compound **444** (Figure 15) demonstrated higher inhibition of PTP1B (IC_50_ = 8.60 ± 0.53 μmol/L), than the reference inhibitor Na_3_VO_4_ (IC_50_ = 18.15 ± 0.15 μmol/L). Additionally, compounds **444**, **445**, and **447** exhibited superior *α*-glucosidase inhibition (IC_50_ = 36.68–87.81 μmol/L) compared to acarbose (IC_50_ = 296.71 ± 10.21 μmol/L). Notably, compounds **446** (IC_50_ = 27.75 ± 3.67 μmol/L) and **447** (IC_50_ = 7.11 ± 0.29 μmol/L) demonstrated significant anti-inflammatory activity, surpassing the positive control indomethacin (IC_50_ = 32.52 ± 2.90 μmol/L) (Dai et al. 2024).

**Figure 16.**
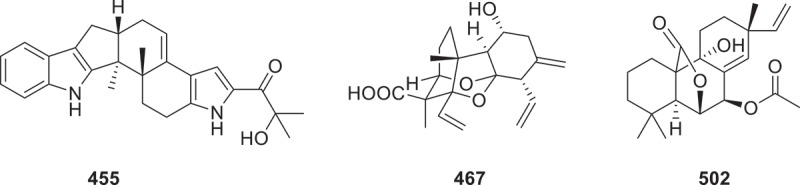
Structures of compounds **455**, **467**, and **502**.

The enzyme TlnA was heterologously expressed in *Aspergillus oryzae* NSAR1, leading to the production of two new products, talarodiene (**451**) and a minor product (452^*^) from the fungus *Talaromyces stipitatus*. These compounds represent a unique cyclohexane-fused 5/8/6 ring system (Li et al. 2024b). Compounds 453^*^ and 454^*^, identified as biosynthetic intermediates of (−)-vinigrol, were isolated from the fungus *Virgaria nigra* NBRC 9238 (Xu and Zou 2024). Through comprehensive chemical investigations, eight novel indole diterpenoids schipenindolenes A–H (**455**–**462**) with diverse skeleton types were isolated from the endophytic fungus *Penicillium* sp. DG23. Compound **455** (Figure 16) showed an ability to degrade HMG-CoA reductase protein, with a half-maximal degradation concentration (DC_50_) of 0.81 μmol/L (Su et al. 2024). A new fusicoccane diterpenoid, harziaderma A (**463**), two novel harziane diterpenoids, harzianones G (**464**) and H (**465**) were isolated from the fungus *Trichoderma harzianum* (Bao et al. 2024a). The new compounds named fusariumic acids A−H (**466**−**473**) were isolated from *Fusarium oxysporum* f. sp. *radicis-lycopersici*. Fusariumic acid B features a novel 7,8-*seco*-isocassadiene scaffold (Figure 16) (Gu et al. 2024b). The combinatorial application of five elicitors with distinct concentrations and induction mechanisms to *Schizophyllum commune* NJFU21 triggered the upregulation of an unprecedented class of linear diterpenoid-derived analogues, including 11 previously undescribed derivatives, schizostatins B–L (**474**–**484**) (Wang et al. 2024k). Eighteen new isopimarane-type diterpenoids, designated as pleosmaranes A−R (**485**−**502**), were isolated from the mangrove endophytic fungus *Pleosporales* sp. HNQQJ-1. Compounds **485**−**493** possess an unusual aromatic B ring and a 20-nor-isopimarane skeleton, while **499**–**501** contain a unique 2-oxabicyclo[2.2.2]octanemoiety. Pleosmaranes R (**502**) features an unexpected 2-oxabicyclo[3.2.1]-octane moiety (Figure 16). Pleosmaranes A (**485**), H (**492**), and L (**496**) demonstrated potent anti-inflammatory activity with IC_50_ values ranging from 19 to 30 μmol/L, exhibiting greater potency than the positive control *L*-NMMA (IC_50_ = 33 μmol/L) (Wang et al. 2024c). From the fungus *Penicillium camemberti* JSB-7212, 12 highly oxygenated novel diterpenes, penicamins A−L (**503**−**514**), were successfully isolated (Pei et al. 2024).

From the mangrove-sediment-derived fungus *Penicillium* sp. SCSIO 41411, two new compounds peniditerpenoids A (**515**) and B (**516**) were obtained. Compound **516** was the first reported *N*-oxide derivative of indole diterpenoid (Cai et al. 2024a). Three new fusidane-type nortriterpenoids, simplifusinolide A (**517**), 24-*epi* simplifusinolide A (**518**), and simplifusidic acid L (**519**), were isolated from the marine-derived fungus *Simplicillium lamellicola* (Kwon et al. 2024). Five new compounds (**520**^#^–**524**^#^) are identified from *Humicola fuscoatra* NRRL 22980. These compounds belong to the triterpenoid carbon framework with a unique *E*-ring-cleaved fernane triterpene skeleton (Cao et al. 2024). The heterologous expression of BGCs from *Aspergillus homomorphus*, *A. fumigatus*, and *A. alliaceus* in *A. oryzae* enabled the discovery of novel onoceroid triterpenoids, homomonoceroid A (**525**) from the former strain, fumionoceroids A–C (**526**–**528**) from the second, and alliaonoceroids B–D (**529**–**531**) from the latter (Tang and Matsuda 2024). The phytochemical study of the *Pisolithus arhizus* fruiting body methanol extract led to the isolation of six new triterpenoids (**532**−**537**) (Parisi et al. 2024). Six new compounds, neodidymelliol A (**538**), and neodidymellioic acids A−E (**539**−**543**) were isolated from the fungus *Neodidymelliopsis negundinis* (Pripdeevech et al. 2024).

**Figure 17.**
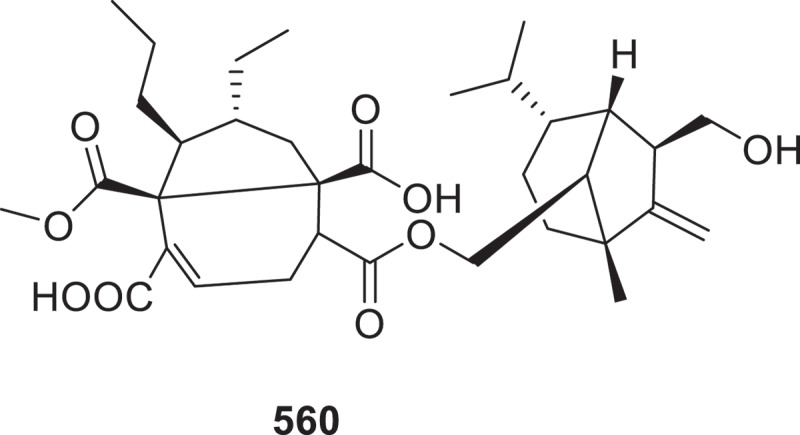
Structure of compound **560**.

**Figure 18.**
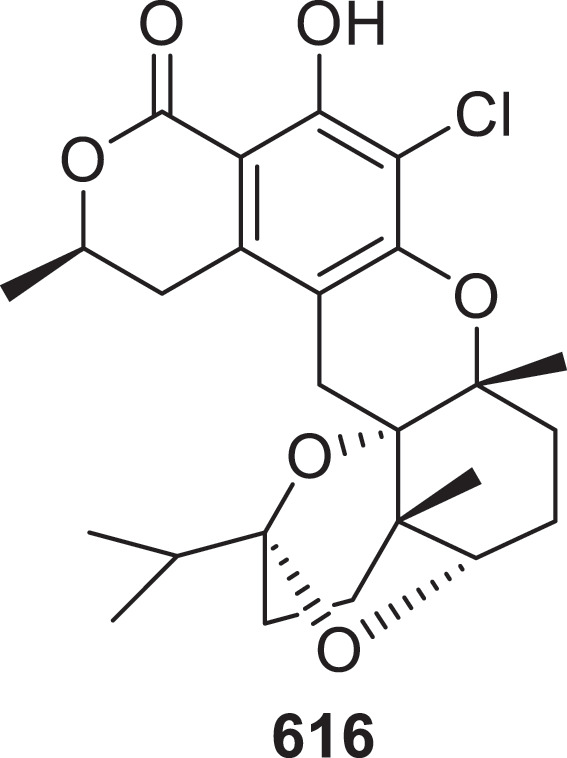
Structure of compound **616**.

The new compounds bialorastins A–F (**544**–**549**) were isolated from the marine-derived fungus *Penicillium bialowiezense* CS-283. Compound **544** represents a rare 17-nor-andrastin that possesses an unusual 2-oxaspiro [4.5]decane-1,4-dione moiety with a unique 6/6/6/6/5 polycyclic system (Yang et al. 2024b). Three new meroterpenoids, bioaspermeroterpenoids A–C (**550**–**552**), were generated through biotransformation of aspermeroterpene C by *Penicillium herquei* GZU-31–6, with **550** being the first meroterpenoid featuring a novel hexadecahydroacephenanthrylene scaffold. Compound **552** exhibited significant anti-inflammatory activity by inhibiting nitric oxide production with an IC_50_ value of 7.50 μmol/L, surpassing the positive control indomethacin (IC_50_ = 24.1 μmol/L) (Yan et al. 2024a). The fungus *Penicillium herquei* GZU-31-6 yielded penihemeroterpenoids A–C (**553**–**555**), pioneering meroterpenoids featuring a novel 6/5/6/5/5/6/5 heptacyclic scaffold, alongside their biosynthetic precursors penihemeroterpenoids D–F (**556**–**558**) (Deng et al. 2024a). From the endophytic fungus *Bipolaris victoriae* S27, 12 unprecedented terpene-nonadride heterodimers bipoterprides A–L (**559**–**570**) and the monomeric compound bipolenin O (**571**) were isolated. Notably, compounds **559**–**570** represent the first discovery of hybrid molecules combining terpene and nonadride units in nature. Additionally, the nonadride moieties in compounds **559** and **560** (Figure 17) feature a unique 5/6 bicyclic framework, marking the first reported cases of such structures in natural nonadrides. Compound **560** exhibited strong inhibitory effects against colorectal cancer (CRC) cell lines (HCT116, SW480, HT29, RKO, and SW620), with MIC values ranging from 3.37 to 17.03 μmol/L. Its potency was comparable to the first-line clinical agent cisplatin (MICs = 14.22–24.31 μmol/L) and exceeded that of 5-Fluorouracil (MICs = 39.12–326.93 μmol/L) (Feng et al. 2024).

(±)-Penindolenes A–D (**572a/b**–**575a/b**), the first representatives of indole terpenoids featuring a *γ*-lactam skeleton, were isolated from the mangrove-derived endophytic fungus *Penicillium brocae* MA-231. In the acetylcholinesterase inhibitory assay, **572a** demonstrated superior inhibitory activity (IC_50_ = 0.098 μmol/L) to tacrine (IC_50_ = 0.10 μmol/L) which served as the positive control (Meng et al. 2024a). A metabolomic exploration of *Armillaria ostoyae* uncovered three unprecedented melleolide dimers bismelleolide BH (**576**), EH (**577**), and CH (**578**), along with melleolide linoleate (**579**), armillarine linoleate (**580**) and melleolide H linoleate (**581**) decorated with fatty acid substituents. The investigation further identified 11 chemically distinct sesquiterpene aryl esters (**582**^#^–**592**^#^) (Pfütze et al. 2024). New Dhilirane-type meroterpenoids dhilirolides O−U (**593**−**599**) were identified from Antarctic fungus *Penicillium purpurogenum* AN13. The unprecedented 6/6/6/6/5/5/6 scaffold configuration is uniquely exhibited by compounds **593** and **594** (Sun et al. 2024d). Fourteen previously undescribed sesterterpenoids maydisens A−N (**600**−**613**) were isolated from *Bipolaris maydis*. For the first time in *Bipolaris maydis*, compounds **600**–**604** were found to contain a structurally rare 5/11 fused bicyclic framework (Shen et al. 2024c). The C-14 epimer of (−)-guignardone I (**614**) is rare in this type of meroterpenoid. It was isolated from the forest endophytic fungus *Phyllosticta* sp. YCC4 (Díaz et al. 2024). From the desert soil-derived fungus *Talaromyces pinophilus* LD-7, 10 new drimane meroterpenoids, named talarines A–J (**615**–**624**), were isolated. Compounds **616** (Figure 18) and **624** displayed potent antiviral activity against vesicular stomatitis virus, with IC_50_ values of 18 nmol/L and 15 nmol/L, respectively, surpassing the efficacy of the positive control ribavirin (IC_50_ = 46 nmol/L) (Ren et al. 2024). The novel compound 12*S*-aspertetranone D (**625**) was isolated from the deep-sea fungus *Aspergillus* sp. SY2601, a strain associated with the Mariana Trench (Sun et al. 2024b). Co-cultivation of the fungi *Alternaria brassicicola* and *Penicillium* sp. HUBU0120 resulted in the discovery of granulathiazole A (**626**), a structurally rare benzothiazole-integrated meroterpenoid (Kong et al. 2024). Three new eremophilane sesquiterpenoids paraphaterpene E–G (**627**–**629**) were isolated from *Paraphaeosphaeria* sp. (Liao et al. 2024). Utilising molecular networking coupled with bioactivity screening, three new polycyclic tetramic acid derivatives (**630**–**632**) with *cis*-decalin systems were isolated from *Epicoccum* sp. 1-042, designated as epicolidines A–C (Chang et al. 2024b).

### 2.3. Steroids

Steroids are a class of natural organic compounds characterised by a four-ring cyclopentano-perhydrophenanthrene carbon skeleton. They are widely found in nature and possess diverse biological activities, including roles in hormone regulation, anti-inflammatory effects, cell signalling, etc. Fungal steroids, in particular, are known for their unique structures and potential pharmacological properties. This section reviews 28 new steroids derived from fungi in 2024.

**Figure 19.**
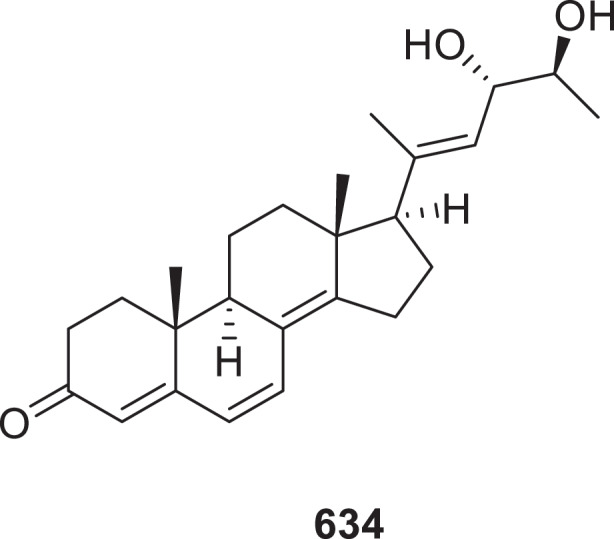
Structure of compound **634**.

Five novel 23,24-diols containing ergosterols, named citristerones A−E (**633**−**637**) were isolated from the endophytic fungus *Penicillium citrinum* TJ507. Compound **634** (Figure 19) demonstrated more potent anti-neuroinflammatory effects in LPS-stimulated BV-2 microglia, suppressing NO production (IC_50_ = 0.60 ± 0.04 μmol/L) than dexamethasone (IC_50_ = 8.03 ± 0.70 μmol/L) (Zhang et al. 2024e). From *Penicillium herquei*, two new [4 + 2] Diels-Alder cycloaddition ergosteroids penicimides A and B (**638** and **639**) and three previously undescribed oxidised ergosteroids peniciroids A−C (**640**−**642**) were isolated. Notably, compounds **638** and **639** are the first reported cycloadducts formed between a steroid and either 1,4,6-trimethyl-1,6-dihydropyridine-2,5-dione or 4,6-dimethyl-1,6-dihydropyridine-2,5-dione. In the evaluation of LPS-triggered NO inhibition using RAW264.7 cells, compound **640** displayed potent anti-inflammatory activity with an IC_50_ value of 7.37 ± 0.69 μmol/L, surpassing the efficacy of dexamethasone (IC_50_ = 8.03 ± 0.70 μmol/L) (Deng et al. 2024b). Three new ergosterol derivatives brassisterols A−C (**643**−**645**) were isolated from the co-cultivation of *Alternaria brassicicola* and *Penicillium granulatum* (Tong et al. 2024).

Three previously unknown steroids hertramycines J−L (**646**−**648**) emerged through cross-induction between co-cultured *Herpotrichia* sp. SF09 and *Trametes versicolor* SF09A (Wang et al. 2024o). Phomosterol C (**649**), a previously unreported ergostane steroid, emerged from symbiotic cultivation of *Phomopsis asparagi* DHS-48 with *Phomopsis* sp. DHS-11 (Wu et al. 2024b). From the deep-sea fungus *Aspergillus terreus* YPGA10, 10 novel ergone derivatives (**650**^#^−**659**^#^) were identified. These compounds share a common structural feature represented as a highly conjugated 25-hydroxyergosta-4,6,8(14),22-tetraen-3-one core (Zhang et al. 2024g). Aoergostane (**660**) is a novel ergostane-type steroid with the *β*-lactone moiety, discovered from the co-culture of *A. oryzae* and *Epicoccum dendrobii* (Shen et al. 2024a).

### 2.4. Alkaloids

Following the detailed discussion of polyketides, terpenoids, and steroids, this section focuses on alkaloids, another major class of fungal secondary metabolites. Alkaloids are a diverse group of naturally occurring organic compounds containing nitrogen atoms. They are predominantly found in plants, fungi, and some animals (Ferreira 2022). This section introduces 108 alkaloid compounds derived from fungi in 2024.

A novel hybrid compound, stephaochratidin A (**661**), was discovered from the deep-sea-derived fungus *Aspergillus ochraceus* MCCC 3A00521 (Zou et al. 2024). Two novel polyketide-peptide hybrid alkaloids, named penisimplicins A (**662**) and B (**663**), were isolated from the fungus *Penicillium simplicissimum* JXCC5. The molecular architectures of compounds **662** and **663** demonstrate a novel mode of covalent linkage, where amino acid-derived units are directly fused to a polyketide framework through carbon-carbon bonding, a configuration not previously documented in natural product chemistry. Notably, penisimplicin A (**662**) demonstrated potent acetylcholinesterase inhibitory activity with an IC_50_ value of 6.35 mmol/L (Wang et al. 2024e). Six new piperazine alkaloids, arizonamides A and B (**664** and **665**), arizolidines A and B (**666** and **667**), and chrysosporazines V and W (**668** and **669**), featuring unique heterobicyclic and spirocyclic isoquinolone skeletal frameworks were discovered from *Penicillium arizonense* CBS 141311.14 through integrated genomic analysis and heterologous biosynthesis strategies. The biosynthetic pathway of compounds **664**−**669** involves two key non-heme iron enzymes, ParF and ParG (Pham et al. 2024). Seven novel alkaloids (**670**^#^−**676**^#^) were isolated from the solid fermentation of *Aspergillus fumigatus* VDL36 (Wang et al. 2024g).

**Figure 20.**
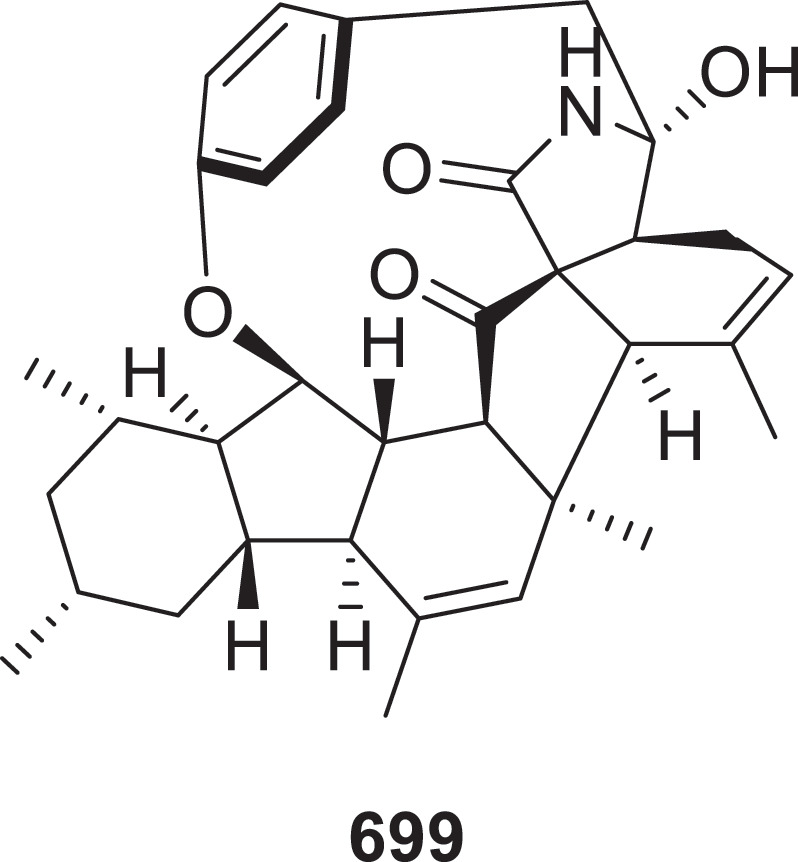
Structure of compound **699**.

The medicinal mushroom *Hericium erinaceus* is also a major source of novel natural products from fungi (Wei et al. 2023). Twelve novel isoindolin-1-one derivatives, named erinacenones A−L (**677**−**688**), were isolated from liquid cultures of the medicinal mushroom *Hericium erinaceus* (Yuan and Liu 2024). Supplementation of *Nigrospora chinensis* GGY-3 cultures with 1-methyl-L-tryptophan (1-MT) led to the biosynthesis of six novel compounds, nigroindolones A−C (**689**−**691**) and racemic (±)-nigroindolones D−F (**692a**/**b**−**694a**/**b**) (Wang et al. 2024a). Four new *γ*-lactam alkaloids, talaroilactams A−D (**695**−**698**), were identified from the fermentation products of the fungus *Talaromyces hainanensis* sp. nov. (Shi et al. 2024d). Eight new decahydrofluorene-class alkaloids, microascones A (**699**) and B (**700**), 2,3-epoxyphomapyrrolidone C (**701**), 14,16-epiascomylactam B (**702**), 24-hydroxyphomapyrrolidone A (**703**), and microascones C−E (**704**−**706**) were isolated from the marine-derived fungus *Microascus* sp. SCSIO 41821. Compounds **699** (Figure 20) and **700** feature unprecedented complex macrocyclic alkaloid skeleton with a 6/5/6/5/6/5/13 polycyclic system. With MICs between 0.10 and 0.80 μg/mL against the seven pathogenic bacteria including *Bacillus amyloliquefaciens*, *B. subtilis*, *Escherichia coli*, *Staphylococcus aureus*, MRSA, *S. agalactiae*, and *S. iniae*, compound **702** displayed more potent antimicrobial activity than the reference drugs gentamicin sulphate and vancomycin (Yao et al. 2024).

Four new alkaloid derivatives, penilactams A (**707a**/**b**), B (**708**), and C (**709**), are synthesised via crosstalk between two pathways mediated by the *pem* gene cluster in *Penicillium* (Li et al. 2024e). Sydowimide A (**710a/b**) was isolated from the marine fungus *Aspergillus sydowii* DL1045 (Shi et al. 2024b). Three new indolyl diketopiperazine compounds, named asperindopiperazines A−C (**711**−**713**), were isolated and identified from the Mariana Trench-associated fungus *Aspergillus* sp. SY2601 (Sun et al. 2024b). Nine novel sesquiterpene alkaloids, designated as eurochevalierines A−I (**714**−**722**), were isolated from crude extract of the endophytic fungus *Penicillium* sp. HZ-5. Screening for antitumor activity revealed that eurochevalierine A (**714**) effectively inhibited the proliferation of triple-negative breast cancer (TNBC) cells (IC_50_ = 8.5 μmol/L) and induced apoptosis, with superior efficacy compared to docetaxel control (IC_50_ = 9.2 μmol/L) (Zhang et al. 2024f). Four unique phenylhydrazone alkaloids, named talarohydrazones A−D (**723**−**726**), were obtained from the deep-sea cold seep fungus *Talaromyces amestolkiae* HDN21-0307. Compound **723** features an uncommon structure integrating 2,4-pyridinedione with phenylhydrazone. Compound **724** is the first natural product that combines a phenylhydrazone with an azadophilone scaffold (Wu et al. 2024a).

**Figure 21.**
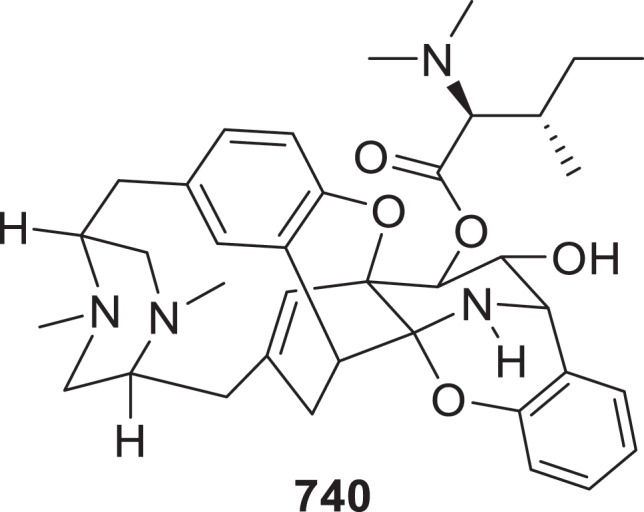
Structure of compound **740**.

Eleven xanthone-based alkaloids were isolated and identified as sydoxanthones F (**727a**/**b**), G (**728**), H (**729a**/**b**), I (**730**), J (**731a**/**b**), and K−M (**732**−**734**) from the marine-derived fungus *Aspergillus sydowii* (Ge et al. 2024). Four unprecedented adenine-polyketide hybrids named ogersonins C−F (**735**−**738**) were isolated from *Clonostachys rogersoniana* (Hou et al. 2024). One novel compound 4’-phosphomuscarine (**739**) was isolated from the mycelium of the poisonous mushroom *Clitocybe rivulosa*. Compound **739** is a phosphorylated derivative of muscarine, with a phosphate group (-PO_3_H_2_) attached to the C-4’ position of muscarine (Dörner et al. 2024). The characterisation of a conserved biosynthetic gene cluster (BGC) from the fungus *Aspergillus parvulus* NRRL 4753 resulted in the isolation of octacyclin A (**740**), an octacyclic natural product with a unique structure that includes two *iso*[3.3.1]bicycles, as well as a combination of fused, bridged, and macrocyclic rings (Figure 21). The complete biosynthetic pathway of octacyclin A (**740**) was elucidated via heterologous expression and enzymatic assays. Initiated by two *L*-tyrosine and one *L*-phenylalanine units, three single-module NRPS enzymes (OcyF, OcyN, OcyD) sequentially activate and assemble the scaffold. Critically, a unique domain in OcyN catalyses unconventional C–C/C–N bond formation to yield a tetrahydroquinolinone intermediate. Two P450 enzymes (OcyM, OcyL) then drive tandem cyclisation through aryl-ether bonds, constructing the unprecedented octacyclic core (Zhang et al. 2024d). Asperalins G (**741**) and H (**742**) were isolated from the marine-derived fungus *Aspergillus alabamensis* SYSU-6778 (Zeng et al. 2024b). A novel alkaloid phomopyrazine (**743**) was successfully isolated through a co-cultivation approach involving two endophytic fungal strains, *Phomopsis asparag* DHS-48 and *Phomopsis* sp. DHS-11 (Wu et al. 2024b).

Two novel prenylated indole 2,5-diketopiperazine alkaloids, named brevianamides E1 (**744**) and E2 (**745**), were obtained from fungus *Penicillium*. Upon studying the degradation of brevianamides E2 (**745**) in acidic methanol, a new methoxylated derivative (746^*^) and two new ring-opened products (747^*^ and 748^*^) were discovered. These latter compounds feature a rearranged and elongated 4-methylpent-3-ene side chain (Khong et al. 2024). Through bioassay-guided fractionation, a new analogue of akanthomycin (**749**) was isolated from *Mycoplasma genitalium* (Peramuna et al. 2024). Integrated genome mining and heterologous biosynthesis strategies enabled the discovery of 10 novel indole-piperazine alkaloids campesines A−G (**750**, **751**, **752a**/**b**−**754a**/**b**, **755**, and **756a/b**) from *Aspergillus campestris* IBT 28561. Significantly, campesines E−G feature an unprecedented octacyclic system (6/5/6/6/6/6/5/6) formed through strategic C-C/C-N bond formation (He et al. 2024).

### 2.5. Peptides

**Figure 22.**
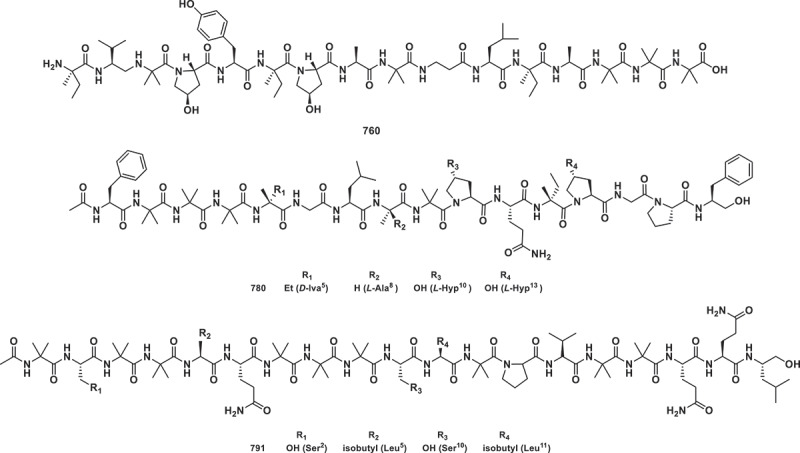
Structures of compounds **760**, **780**, and **791**.

This section documents 75 novel fungal-derived peptides reported in 2024, encompassing peptaibols (short-chain polypeptides), cyclic peptide scaffolds, piperazine-containing compounds, and structurally diverse analogues. A bioassay-guided investigation of the deep-sea-derived fungus *Simplicillium obclavatum* EIODSF 020 led to the discovery of 11 novel peptaibiotics, simplicpeptaibs A−K (**757**−**767**). Compounds **760** (Figure 22) and **762** displayed enhanced activity against the tobacco pathogen *Ralstonia solanacearum* (24 h MIC_90_ peptides = 25.0 μg/mL), surpassing the positive drug streptomycin sulphate (24 h MIC_90_ = 50 μg/mL) (Liang et al. 2024). In addition to antibacterial agents, cytotoxic peptaibols have emerged as potential anticancer leads. Twelve novel peptaibols, trichoguizaibols A−L (**768**−**779**), isolated from an urban soil-derived fungus *Trichoderma guizhouense* DS9-1, exhibited IC_50_ values (0.68−4.5 μmol/L) against MDA-MB-231, SK-Hep1, SKOV3, DU145, and HCT116 cells. Although less potent than docetaxel (IC_50_ = 0.004−0.17 μmol/L), these compounds represent a valuable scaffold for further structural optimisation (Han et al. 2024a). Similarly, antiamoebins XVII–XXI (**780**−**784**) from *Emericellopsis* sp. XJ1056, inhibiting *Amaranthus retroflexus* radicle growth (IC_50_ = 19.7 μg/mL), outperforming the known herbicide glyphosate (IC_50_ = 49.5 μg/mL) as positive control (Song et al. 2024). Five new lipopeptaibols lipostrigaibols A−E (**785**−**789**) and eight new 19-residue peptaibols strigaibols A−H (**790**−**797**) were isolated from a soil-derived fungus *Trichoderma strigosum*. Compounds **791**, **792**, **794**, and **797** were significantly more active than the positive control (doxorubicin, IC_50_ = 71.4 ± 13.4 − 91.2 ± 27.6 μmol/L) on the cytotoxicity against MDA-MB-231, SNU449, SKOV3, DU145, and HCT116 cancer cell lines, with IC_50_ values ranging from 1.0 ± 0.1 to 13.0 ± 0.6 μmol/L (Park et al. 2024).

Violaceotides B−E (**798**−**801**) were four newly discovered cyclic tetrapeptides extracted from cultures of *Aspergillus insulicola* IMB18-072 co-cultured with *Alternaria angustiovoidea* IMB20-805 (Li et al. 2024d). Two unprecedented chlorinated cyclotetrapeptides named omnipolyphilins A−B (**802**−**803**) were isolated from *Polyphilus sieberi* (Wennrich et al. 2024a). Molecular networking-guided isolation of the Mariana Trench anemone derived fungus *Hamigera ingelheimensis* MSC5 resulted in the separation of 12 novel cyclic pentapeptides, avellanins D−O (**804**−**815**). Among them, bioassay demonstrated that compound **802** exhibited potent antimalarial activity (IC_50_ = 0.190 ± 0.090 μmol/L), with artemisinin (IC_50_ = 0.010 ± 0.002 μmol/L) and chloroquine (IC_50_ = 0.032 ± 0.005 μmol/L) as positive controls (Li et al. 2024c). Two cycloheptapeptides, scortides A and B (**816** and **817**), were isolated and identified from the sea-anemone-derived fungus *Talaromyces scorteus* AS-242. It is worth noting that compound **817** showed significant activities against the pathogenic fungus *Curvularia spicifera* with MIC value of 1 μg/mL, while the positive control (amphotericin B) showed MIC values of 0.5 μg/mL (Wang et al. 2024m). New proline-containing compounds endolides E (**818**) and F (**819**), two cyclic peptides, were isolated from the marine sponge-derived fungus *Stachylidium bicolor* 293 K04 (Berger et al. 2024).

Genome mining strategy and heterologous expression of dimethylallyl tryptophan synthase (DMATS) gene clusters that were refactored with highly inducible promoters in *Aspergillus nidulans* CBS 101889 yielded six prenylated diketopiperazines, homomorphins A−F (**820**−**824**), marking the first discovery of C_4_-prenylated tryptophan-containing diketopiperazines and their derivatives (Jenkinson et al. 2024). Cycloaspeptide H (**825**), a novel cyclopentapeptide, was isolated from *Penicillium virgatum* GDGJ-227 (Li et al. 2024a). Concurrently, cotteslosin D was characterised as a new cyclopentapeptide from *Aspergillus versicolour* 2-18 (Zheng et al. 2024b). Through genome mining and heteroexpression, biosynthetic gene cluster (*hvm*) for the sterol *O*-acyltransferase inhibitor helvamide from the genome of *Aspergillus rugulosus* MST-FP2007 was successfully identified and activated. Heterologous expression of the *hvm* cluster in the *Aspergillus nidulans* led to the isolation of an unreported phenylpropanoid piperazines helvamide B (**827**) (Wang et al. 2024f). From the metabolites produced by the fungus *Talaromyces* sp. SCSIO 41412 isolated from the mangrove sediment, four new *p*-terphenyl derivatives talaroterphenyls A−D (**828**−**831**) were identified (Cai et al. 2024b).

### 2.6. Glycosides

Glycosides, also known as glycosyl derivatives, are generally formed by the reaction between the hemiacetal hydroxyl group of a monosaccharide and the hydroxyl group of an alcohol or phenol, resulting in the loss of water to form an acetal derivative (Santos et al. 2021). Fungal glycosides are classified by the biosynthetic origin of their aglycone and their glycosidic linkage type (C-, O-, or *N*-). Unlike their plant counterparts, fungal glycosides frequently contain rare deoxy sugars and exhibit broader bioactivities, stemming from their unique structural modifications. This section reviews 50 new glycosides derived from fungi in 2024.

Twenty novel aromatic polyketide C/O glycosides were isolated from the fungi *Penicillium chlamydospora, Trichoderma cellulolyticus*, and *Verticillium dahliae*. They were identified as talarocellmycins A−I (**832**−**841**), phaeomoniecins A−H (**842**−**849**), verapyrone A (**850**), and verapyrone B (**851**). Compounds **843**−**847** displayed anti-HIV activity with IC_50_ of 2.3−8.6 µmol/L (Chen et al. 2024a). Three novel *α*-pyrone-polyketide glycosides, proliferapyrone A (**852**), proliferapyrone C (**853**), and proliferapyrone D (**854**) were produced as secondary metabolites by the enzyme system encoded by the gene cluster of the fungus *Fusarium proliferatum* (Wang et al. 2024h). Luteodienoside A (**855**) is a newly discovered glycosylated polyketide originating from the fungus *Aspergillus luteorubrus* MST-FP2246. Luteodienoside B (**856**) is a regioisomer generated during the heterologous expression of the *ltb* gene cluster in *Aspergillus nidulans* (Arishi et al. 2024). Through metabolic genomics mining, seven *O*-glycosylated depsidones compounds named exopxenmycins A−G (**857**−**863**) were discovered in the marine-derived fungus *Exophiala xenobiotica* SDU 039 (Yang et al. 2024a).

**Figure 23.**
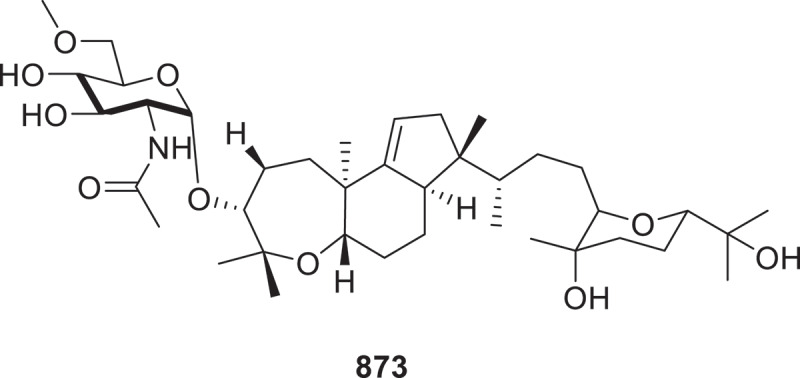
Structures of compound **873**.

When the isopod-associated fungi *Herpotrichia* sp. SF09 and *Trametes versicolor* SF09A were co-cultured, they induced the formation of two new compounds, hertramycines A and B (**864** and **865**) (Wang et al. 2024o). Through genome mining and synthetic biology strategies, the *res* gene cluster from *Aspergillus sclerotiorum* LZDX-33-4 was shown to drive biosynthesis of epipyrone A (**866**), a novel hybrid polyketide incorporating structural moieties from restricticin and cichorine (Fan et al. 2024a). Biosynthetic exploration of the fungal consortium comprising *Clonostachys* sp. S4S-07771A07 and *Coccidioides* sp. S4S-14879B01 yielded four novel triterpenoid amide glycosides, designated pullenvalenes E−H (**867**−**870**), through targeted co-culture induction (Wang et al. 2024l). Two new nucleoside derivatives helicosides D and E (**871** and **872**), were isolated from the fungus *Helicoma septoconstrictum* (Zheng et al. 2024c). Four new compounds, named pullenvalenes A−D (**873**−**876**), were isolated from the strains *Talaromyces* sp. CMB-TN6F and *Coccidioides* sp. CMB-TN39F (Figure 23), together with compounds **874**−**876** exhibit a novel triterpenoid carbon framework that has not been previously characterised, coupled with uncommon glycosidic moieties derived from 6-*O*-methyl-*N*-acetyl-D-glucosamine (Dewa et al. 2024). Three previously undescribed glycosides (**877**^#^−**879**^#^) were isolated and characterised from the fungal species *Humicola fuscoatra* NRRL 22980 (Cao et al. 2024). Two novel glycosidic compounds, neodrymelliosides A (**880**) and B (**881**), were obtained from the fungal species *Neodidymelliopsis negundinis* (Pripdeevech et al. 2024).

## 3. Discussion and conclusions

Fungal-derived natural products continue to demonstrate remarkable chemical diversity and medicinal potential, underscoring their significance in drug discovery and biotechnology. Based on our analysis, we have identified a series of compounds demonstrating remarkable *in vitro* activities. Notable examples include the potent antifungal compounds **21**^#^−**34**^#^, the cytotoxic agent **248**, and the antiviral compound **624**. These findings collectively highlight the significant potential of fungal-derived secondary metabolites in therapeutic applications against infectious diseases and other medical conditions.

**Figure 24.**
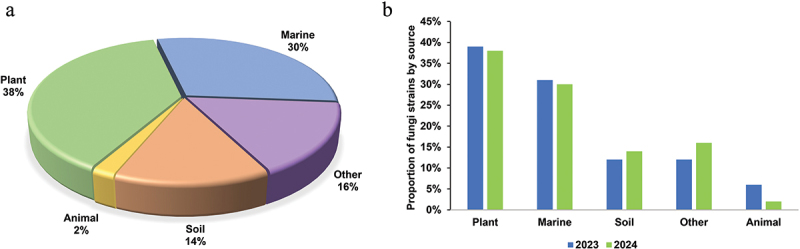
Number of novel fungal compounds in 2024. (a) Divided by sources of fungal strains. (b) Comparison of fungal sources between 2023 and 2024.

Despite persistent challenges, including low yields, structural complexity, and unresolved bioactivity mechanisms, advances in fungal natural product research have expanded our understanding of their biosynthetic pathways and therapeutic applications. Ecological source analysis revealed that plant-associated fungi (38%) and marine-derived strains (30%) dominated the isolation of novel compounds in 2024, collectively accounting for two-thirds of discoveries, while soil (14%) and animal-associated fungi (2%) represented smaller proportions (Figure 24a). This distribution pattern aligns closely with 2023 data, indicating stable ecological preferences in compound production (Figure 24b).

**Figure 25.**
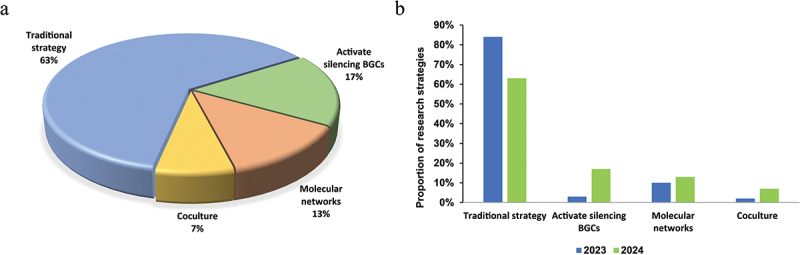
Number of novel fungal compounds in 2024. (a) Divided by research strategies. (b) Comparison of research strategies between 2023 and 2024.

The exploration of bioactive secondary metabolites from extensive fungal resources for pharmaceutical applications presents a daunting challenge, particularly in structurally characterising unknown compounds owing to their intricate chemical diversity and trace quantities. Recent advancements in high-throughput untargeted metabolomics, however, have catalysed the evolution of bioinformatics and cheminformatics tools, offering innovative strategies for natural product discovery. As illustrated in Figure 25a, contemporary approaches such as molecular networking, co-cultivation systems, and silent BGCs activation have demonstrated promising potential. Despite these developments, traditional methodologies remain the primary source of novel compound identification, accounting for over 60% of discoveries (Figure 25a). From our perspective, key barriers include computational resource limitations for AI-based BGC prediction, specialised training needs for CRISPR-mediated activation and high costs of synthetic biology workflows.

Statistical trends reveal a notable shift: while molecular networking, co-culture techniques, and silent BGC activation have exhibited progressive adoption since 2023, the reliance on conventional strategies has gradually declined (Figure 25b). This divergence underscores the growing limitations of traditional workflows, yet their continued dominance highlights their irreplaceable role in current research frameworks. Emerging technologies are poised to complement these established methods, potentially unlocking a broader spectrum of fungal-derived chemical entities.

**Figure 26.**
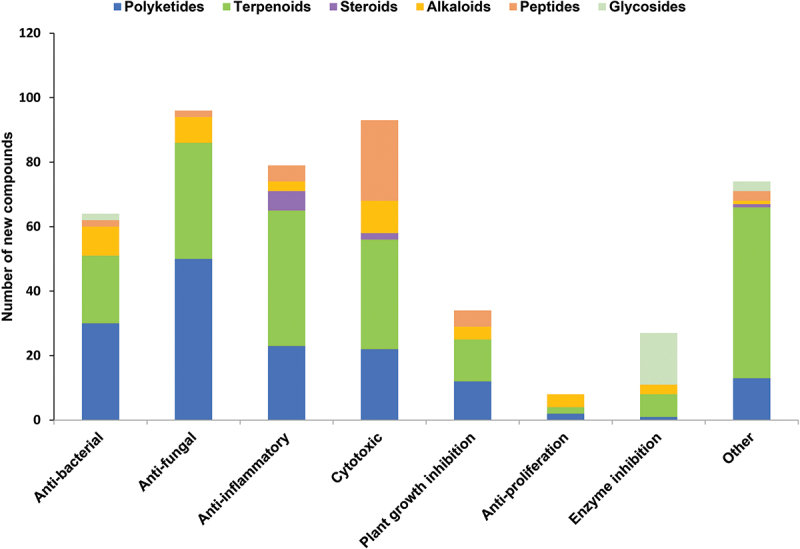
Number of novel fungal compounds in 2024, categorized by bioactivity and structural class.

The year 2024 witnessed the isolation of 907 novel natural product-derived compounds, with close to half (475 compounds, 52.3%) exhibiting pharmacological potential. This statistic underscores a persistent challenge in natural product drug discovery, where the limited yields and structural complexity of bioactive metabolites often hinder their viability as therapeutic candidates. Over the past year, research priorities have centred on evaluating bioactivities such as antimicrobial, antifungal, cytotoxic, anti-inflammatory, plant growth inhibitory, and enzyme inhibitory properties (Figure 26). Notably, compounds with antifungal and cytotoxic functions dominated the dataset, while anti-inflammatory agents followed in prevalence. Further analysis of activity-structure relationships revealed distinct trends: terpenoids were predominantly associated with anti-inflammatory effects, whereas polyketides emerged as the most prolific class exhibiting antifungal activity. These findings highlight both the diversity of fungal-derived metabolites and the need for advanced strategies to optimise their therapeutic exploitation.

In this review, 907 new fungal natural products reported in 2024 were characterised, including 284 polyketides, 362 terpenoids, 108 alkaloids, and smaller proportions of steroids, peptides, and glycosides. These compounds exhibit diverse bioactivities, ranging from antimicrobial and anti-fungi properties to enzyme inhibition, highlighting their potential as leads for pharmaceutical development. Moving forward, integrating multi-omics approaches, synthetic biology, and targeted ecological sampling will be critical to overcoming existing limitations and unlocking the full therapeutic potential of fungal natural products.

## Supplementary Material

Supplemental_material.docx
