# Supplementary material for: New bioactive secondary metabolites from fungi: 2024
Source: Mycology. 2025 Jul 11;16(3):961–87. doi: 10.1080/21501203.2025.2526772 (PMC12422058; doi:10.1080/21501203.2025.2526772)
Supplement: Supplemental_material.docx [file TMYC_A_2526772_SM4583.docx]

Supplemental material

**New bioactive secondary metabolites from fungi: 2024**

Meiyan Bao^a,b,#^, Ying Shi^a,b,#^, Xiaoyi Gong^a^, Yutong Guo^a^, Jing Wang^a^, Xiaofei Chen^a^ and Ling Liu^a,b,*^

^a^State Key Laboratory of Microbial Diversity and Innovative Utilization, Institute of Microbiology Chinese Academy of Sciences, Beijing, China

^b^University of Chinese Academy of Sciences, Beijing, China

^#^These authors contributed equally to this work.

*Corresponding author:

Ling Liu [liul@im.ac.cn](mailto:liul@im.ac.cn)

State Key Laboratory of Microbial Diversity and Innovative Utilization, Institute of Microbiology Chinese Academy of Sciences, Beijing 100101, China

**Table of Contents**

| 1. **Supplementary Figure** |  |
| --- | --- |
| \| **Figure S1.** The unnamed structures in the main text. \| \| --- \| | S1 |
| 1. **Supplementary Table** |  |
| **Table S1.** Compounds with lengthy names not displayed in the main text (abbreviated labels used). | S2 |
| 1. **Supplementary Table** |  |
| **Table S2.** List of abbreviations. | S3 |
|  |  |
|  |  |
|  |  |
|  |  |
|  |  |
|  |  |
|  |  |
|  |  |
|  |  |
|  |  |
|  |  |
|  |  |
|  |  |
|  |  |
|  |  |

**1. Supplementary Figure**

**Figure S1.** The unnamed structures in the main text.

**2. Supplementary Table**

**Table S1.** Compounds with lengthy names not displayed in the main text (abbreviated labels used).

| Number | Name |
| --- | --- |
| **21^#^** | Terreroide A |
| **22^#^** | 8″-epi-Terreroide A |
| **23^#^** | Terreroide B |
| **24^#^** | 8″-epi-Terreroide B |
| **25^#^** | Terreroide C |
| **26^#^** | Terreroide D |
| **27^#^** | 8″-epi-Terreroide D |
| **28^#^** | Terreroide E |
| **29^#^** | Terreroide F |
| **30^#^** | 8″-epi-Terreroide F |
| **31^#^** | Terreroide G |
| **32^#^** | 8″-epi-Terreroide G |
| **33^#^** | Terreroide H |
| **34^#^** | 8″-epi-Terreroide H |
| **35^#^** | 3,5'-dimethyl-2,3'-oxybiphenyl-1,5,1',2'-tetraol |
| **318^#^** | 9,11-dihydroxy-6-oxodrim-7-ene |
| **319^#^** | albrassitriol |
| **320^#^** | *epi*-albrassitriol |
| **321^#^** | ustusolate A |
| **322^#^** | 2-hydroxy-6-*epi*-albrassitriol |
| **323^#^** | ustusolide E |
| **324^#^** | 6-(1′-carboxy-2′4′6′-trien)-9-hydroxydrim-7-ene-11,12-olide |
| **373^#^** | 15*S*-Hydroxybrevione P |
| **374^#^** | 17-Deacetoxy-17*R*-methoxybrevione E |
| **375^#^** | 17-Deacetoxy-17*S*-methoxybrevione E |
| **376^#^** | 19-Hydroxybrevione I |
| **377^#^** | 1*R*-Hydroxyniveulone |
| **378^#^** | 1*S*,2*S*-Dihydroxyniveulone |
| **379^#^** | (1*R*, 4*S*, 5*R*, 10*S*)-10,13,14-Trihydroxycarota-6,8-diene |
| **380^#^** | (1*R*, 4*S*, 10*S*)-10,13,14-Trihydroxycarota-5,7-diene |
| **520^#^** | 2*α*-hydroxy isomotiol |
| **521^#^** | 19*β*-hydroxy isomotiol |
| **522^#^** | 2*α*, 19*β* dihydroxy-isomotiol |
| **523^#^** | 2*α*-hydroxy ismotiol-19-one |
| **524^#^** | 2-deacetyl-3-deglucopyranosyl-fuscoatroside |
| **582^#^** | 5′-*O*-desmethylarmillaribin |
| **583^#^** | 4-dehydroxyarmillaridin |
| **584^#^** | 4-methoxymelleolide H |
| **585^#^** | 1-hydroarmillaricin |
| **586^#^** | 10-hydroxy-5′-*O*-methylarmillane |
| **587^#^** | 4-dehydroxymelleolide F |
| **588^#^** | 10-ketomelleolide E |
| **589^#^** | 4,10-dehydroxymelleolide I |
| **590^#^** | 4-dehydroxymelleolide I |
| **591^#^** | 10-ketomelleolide I |
| **592^#^** | 4-acetylarmillaridin |
| **650^#^** | (9*R*, 10*R*, 12*R*, 13*R*, 17*R*, 20*R*, 24*R*)-12*β*,25,28-Trihydroxyergone |
| **651^#^** | (9*R*, 10*R*, 12*R*, 13*R*, 17*R*, 20*R*, 24*R*)-15-Oxo-12*β*,25,28-trihydroxyergone |
| **652^#^** | (9*R*, 10*R*, 12*R*, 13*R*, 15*S*, 17*R*, 20*R*, 24*R*)-15*α*-Acetoxy-12*β*,25,28-trihydroxyergone |
| **653^#^** | (9*R*, 10*R*, 12*R*, 13*R*, 15*S*, 17*R*, 20*R*, 24*R*)-15*α*-Methoxy-12*β*,25,28-trihydroxyergone |
| **654^#^** | (9*R*, 10*R*, 12*R*, 13*R*, 15*R*, 17*R*, 20*R*, 24*R*)-15*β*-Methoxy-12*β*,25,28-trihydroxyergone |
| **655^#^** | (9*R*, 10*R*, 12*R*, 13*R*, 15*R*, 17*R*, 20*R*, 24*R*)-12*β*,15*β*,25,28-Tetrahydroxyergone |
| **656^#^** | (9*R*, 10*R*, 13*R*, 15*R*, 17*R*, 20*R*, 24*R*)-15*β*,25,28-Trihydroxyergone |
| **657^#^** | (9*R*, 10*R*, 13*R*, 15*S*, 17*R*, 20*R*, 24*R*)-15*α*,25,28-Trihydroxyergone |
| **658^#^** | (9*R*, 10*R*, 12*R*, 13*R*, 15*S*, 17*R*, 20*R*, 24S)-15*α*-Acetoxy-12*β*,25,26-trihydroxyergone |
| **659^#^** | (9*R*, 10*R*, 13*R*, 17*R*, 20*R*, 24*S*)-25,26-Dihydroxyergone |
| **670^#^** | Fumigaclavine I |
| **671^#^** | Fumigaclavine J |
| **672^#^** | Fumiquinazolinine |
| **673^#^** | 11-Dehyroxypseurotin A |
| **674^#^** | 13-Ethoxycyclotryprostatin A |
| **675^#^** | 13-Dehydroxycyclotryprostatin A |
| **676^#^** | 12*β*-Hydroxy-13-oxofumitremorgin C |
| **877^#^** | 3-*O*-(*β*-D glucopyranosyl)-isomotiol |
| **878^#^** | 3-O-(*β*-D glucopyranosyl)-2α-hydroxy-isomotiol |
| **879^#^** | 3-O-(*β*-D glucopyranosyl)-2α-acetoxy-isomotiol |

**3. Supplementary Table**

**Table S2.** List of abbreviations.

| Abbreviations | Full Name |
| --- | --- |
| **sEPSC** | Spontaneous excitatory postsynaptic currents |
| **MIC** | Minimum inhibitory concentration |
| **MBC** | Minimum bactericidal concentration |
| **NLRP3** | NOD-, LRR- and pyrin domain-containing protein 3 |
| **IC_50_** | Half-maximal inhibitory concentration |
| **LD_50_** | Lethal dose, 50% |
| **EC₅₀** | Half-maximal effective concentration |
| **DC₅₀** | Half-maximal degradation concentration |
| **COLO-201** | Human colorectal cancer cells |
| **PTP1B** | Protein tyrosine phosphatase-1b |
| **HMG-CoA** | 3-hydroxy-3-methyl glutaryl coenzyme A reductase |
| **LPS** | Lipopolysaccharides |
